# Repeated injection of multivitamins and multiminerals during the transition period enhances immune response by suppressing inflammation and oxidative stress in cows and their calves

**DOI:** 10.3389/fimmu.2023.1059956

**Published:** 2023-02-09

**Authors:** Yallappa M. Somagond, Mohanned Naif Alhussien, Ajay Kumar Dang

**Affiliations:** ^1^ Lactation and Immuno-Physiology Laboratory, Indian Council of Agricultural Research (ICAR)-National Dairy Research Institute, Karnal, Haryana, India; ^2^ Reproductive Biotechnology, School of Life Sciences, Technical University of Munich, Freising, Germany

**Keywords:** peripartum cows, micronutrients injection, immune response, oxidative stress, calf health

## Abstract

Periparturient dairy cows undergo major physiological and metabolic changes as well as immunosuppression, associated with decrease in plasma concentrations of various minerals and vitamins. The present study was conducted to investigate effects of repeated injections of vitamins and minerals on oxidative stress, innate and adaptive immune response in periparturient dairy cows and their offspring. Experiment was carried out on 24 peripartum Karan-Fries cows, randomly divided into four groups (n=6): control, Multi-mineral (MM), Multi-vitamin (MV) and Multi-minerals and Multi-vitamin (MMMV). Five ml of MM (Zinc 40 mg/ml, Manganese 10 mg/ml, Copper 15 mg/ml, Selenium 5 mg/ml) and five ml of MV (Vitamin E 5 mg/ml, Vitamin A 1000 IU/ml, B-Complex 5 mg/ml, and Vitamin D_3_ 500 IU/ml) were injected intramuscularly (IM) to the MM and MV groups. MMMV group cows were injected with both. In all treatment groups, injections and blood sampling were carried out on 30^th^, 15^th^, 7^th^ days before and after expected date of parturition and at calving. In calves, blood was collected at calving and on 1, 2, 3, 4, 7, 8, 15, 30 and 45 days post-calving. Colostrum/milk were collected at calving and at days 2, 4, and 8 post-calving. A lower percentage of total neutrophils and immature neutrophils, higher percentage of lymphocytes together with increased phagocytic activity of neutrophils and proliferative capacity of lymphocytes found in blood of MMMV cows/calves. Lower relative mRNA expression of TLRs and CXCRs and higher mRNA expression of GR-α, CD62L, CD11b, CD25 and CD44 found in blood neutrophils of MMMV groups. Total antioxidant capacity was higher, activity of antioxidant enzymes (SOD and CAT), TBARS levels were lower in the blood plasma of treated cows/calves. In both cows/calves, plasma pro-inflammatory cytokines (IL-1α, IL-1β, IL-6, IL-8, IL-17A, IFN-γ and TNF-α) increased, whereas anti-inflammatory cytokines (IL-4 and IL-10) decreased in MMMV groups. Total immunoglobulins increased in colostrum/milk of MMMV injected cows and plasma of their calves. Results indicate that repeated injections of multivitamins and multiminerals to peripartum dairy cows could be a major strategy to improve immune response and decrease in inflammation and oxidative stress in transition dairy cows and their calves.

## Graphical Abstract

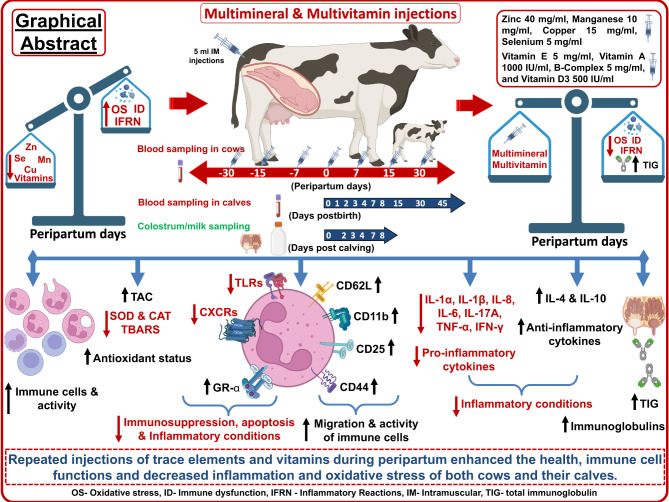

## 1 Introduction

The transition period from 3 weeks before to 3 weeks after calving is the most crucial phase in a cow’s production cycle. During this period, lactogenesis, the regression of the uterus and the accompanying changes in hormone balance and metabolism, present the dairy cow with unique adaptive challenges that are particularly dramatic for the transition dairy cows (1, 2). Most of these changes are associated with inflammatory response induced by various mechanisms governing the production of eicosanoids and proinflammatory cytokines (3–6). Several components of the host defense system are altered, including neutrophil development, migration, and function, lymphocyte proliferation, antibody responses, and cytokine production by immune cells (2, 6, 7). The transition period is also characterized by increased sensitivity of the hypothalamic-pituitary-adrenal (HPA) axis and increased secretion of glucocorticoids, which act as immunosuppressants (8). Immunosuppression during the transition period and the associated high prevalence of diseases severely affect both animal health and productivity and are therefore considered a major challenge for the dairy industry (1, 9). In addition, poor metabolic adaptation, and immune functions of periparturient dairy cows affect the nutritional and immunological properties of the colostrum and ultimately the well-being of the newborn calves (10–12).

High levels of reactive oxygen species (ROS) in periparturient dairy cows could expose them to increased oxidative stress, which contribute to dysfunctional immune responses and increased risk of health disorders in cows and their calves (3). Due to the reduction in feed intake and the simultaneous increased need for antioxidants to counteract the overall increased ROS, supplementation of antioxidants such as vitamins and trace elements to periparturient cows is crucial to minimize the harmful effects of excessive ROS production and thus improve the health status of the animals and reduce the incidence of diseases (13, 14). According to previous reports, a decrease in the concentration of trace elements and vitamins in the blood during the periparturient period can impair many immune functions and lead to various inflammatory diseases in cattle (15–19). Vitamins (A, D, B_12_ and E) and Minerals (zinc, copper, selenium, and iron) are essential for dairy cows’ health and production, as they work as cellular antioxidants and immune system modulators (17, 20). The administration of various trace elements and vitamins to periparturient cows significantly reduces the incidence of various diseases associated with parturition, strengthens the animals’ immune response, and promotes a rapid return to homeostasis (21–23).

One of the major drawbacks of oral intake of micronutrients in cattle is reduced absorption due to interactions with other nutrients at the rumen level (24, 25). For instance, Cope et al. (26) highlighted that Zn deficiency in high-yielding dairy cows is indicative of certain nutritional situations that could affect Zn absorption. Santschi et al. (27) demonstrated that extensive loss of supplemented vitamin B occurs in the rumen of the cows before reaching the small intestine, which reduces the utilization of orally supplemented vitamins. Roughage with higher molybdenum (Mo) and sulphur (S) content leads to the formation of thiomolybdates in the rumen, which bind copper (Cu) ions and form a highly insoluble complex, greatly reducing Cu absorption and utilization (25, 28). In addition, excessive Fe feeding leads to Mn and Cu deficiency in ruminants (24, 29). Also, high Ca, K or P content in the diet increases Mn excretion in the feces, presumably by reducing Mn absorption (24). Therefore, the alternative use of injectable trace micronutrients is an area of great interest. Injectable/parenteral micronutrient supplementation could potentially be an alternative for supplying more micronutrients during the transition period (27, 30, 31). Several studies demonstrated that injection of trace mineral solution could increase the concentration of trace minerals such as Cu and Se in liver for at least 2 weeks post injection in bovines (32, 33). Although the beneficial effects of oral administration of multivitamins and multimineral on production performance and prevention of postpartum diseases in cows have been studied, the relationship between injectable antioxidant micronutrients and immune response in dams and calves is poorly understood. Therefore, the present study was conducted to investigate the effects of repeated injections of vitamins and trace elements on the functions and population of immune cells in the blood, the expression profile of the first cellular line of defense, i.e., neutrophils, oxidative stress, and the concentration of inflammatory cytokines in periparturient dairy cows and their calves.

## 2 Materials and methods

### 2.1 Selection of cows, health and feeding management

The present study was conducted on 24 peripartum healthy Karan Fries (Holstein Friesian X Tharparkar) cows at the Livestock Research Centre of National Dairy Research Institute, Karnal, India, situated 245 m above mean sea level in the bed of Indo-Gangetic alluvial plain, latitude 29° 43’ N and longitude 77° 2’ E. All cows in the experimental groups were high yielders (>10 kg/d) considering the Indian dairy production system which makes them more prone to transition period stress and mammary infection during early lactation as compared to other local breeds (34). The criteria used to form the experimental groups were live weight (approximate of 450 kg), body condition score (average of 3.5) and parity (3^rd^ to 4^th^). The farm’s routine health practices were followed during the experimental period to ensure that all the experimental cows were healthy and free from any pathological, physiological, or infectious disorders. Under the same conditions, all the cows were managed in a properly ventilated stall. The expected day of calving was estimated using accessible data of artificial insemination, which was further confirmed by pregnancy diagnosis. Around seven days before the expected date of calving, the cows were brought to the calving pen and maintained there for four to five days following calving. To meet the nutritional needs of the transition cows, they were fed a total mixed ration (TMR) as detailed in **Table 1**. Detailed information about the chemical composition, premix composition, and negative dietary cation-anion difference (DCAD) are also shown in **Table 1**. The animals had free access to water at all times of the day. The TMR was prepared daily by hand mixing and offered twice at 09:00 AM and 18:00 PM. Both manual and automated milking methods were used to collect milk samples from the experimental cows. During the colostrum phase, hand milking was performed; however, machine milking was used for the rest of the experiment.

**Table 1:** Attributes and chemical composition of total mixed ration (TMR) fed during the experimental period.

Attributes	Content (g/kg DM unless it is mentioned)
Berseem fodder	210
Wheat straw	197
Ground yellow maize	277
Groundnut cake	152
Wheat bran	64
Rice bran	87
Mineral mixture and vitamins premix	10
Salt	3
Chemical composition	
Dry matter	756
Organic matter	856
Crude protein	175
Crude fibre	291
Total ash	95
NDF, neutral detergent fibre	386
ADF, acid detergent fibre	259
Calcium	10
Phosphorus	4.1
Magnesium	2.3
Manganese	63.83 mg/kg
Copper	13.22 mg/kg
Selenium	0.36 mg/kg
Zinc	49.00 mg/kg
β-carotene	51.43 IU/kg
DL-alpha-tocopheryl acetate	60.50 IU/kg

Premix composition per kilogram: Vitamin A 7,50,000 IU, Vitamin D3 220,000 IU, Vitamin E 5000 mg, Cobalt 150 mg, Copper 1200 mg, Iodine 325 mg, Iron 1500 mg, Selenium 54 mg, Magnesium 6000 mg, Manganese 1500 mg, Zinc 9600 mg, DL-Methionine 1000 mg, Calcium 25.50%, Phosphorus 12.75%. Main ionic components: negative dietary cation-anion difference (DCAD) was -24 mEq/100 g of DM during for prepartum period (0.55% Na, 1.48% K, 0.61% Cl, 0.31% S). The following formula was used to calculate DCAD = [(mEq of K) + (mEq of Na)] – [(mEq of Cl) + (mEq of S)].

### 2.2 Management of newborn calves

Calves born to these cows were separated from the dams and housed separately in a calf’s pen after weighing and ear tagging. Each pen had a 1 m^2^ concrete floor covered by an asbestos roof, and a 2 m^2^ open area for loafing. The pens had facilities to protect the calves from cold. Each pen had feed trough and the animals always had free access to water. Before collecting colostrum from dam, teats were cleansed with water, disinfected with hypochlorite, and dried. Within 2 hours of birth, the same dam’s colostrum was fed to the newborn calves *via* nipple bottle, marked as 0 hour, followed by further feedings at a 12-hour interval for five days. Calves received colostrum from their dams at 10% of body weight, regardless of the quality of colostrum. The newborn calves were given milk at the rate of 1/10^th^ of their body weight from 5 days of birth until the completion of the trial.

### 2.3 Experimental design

**Figure 1:**
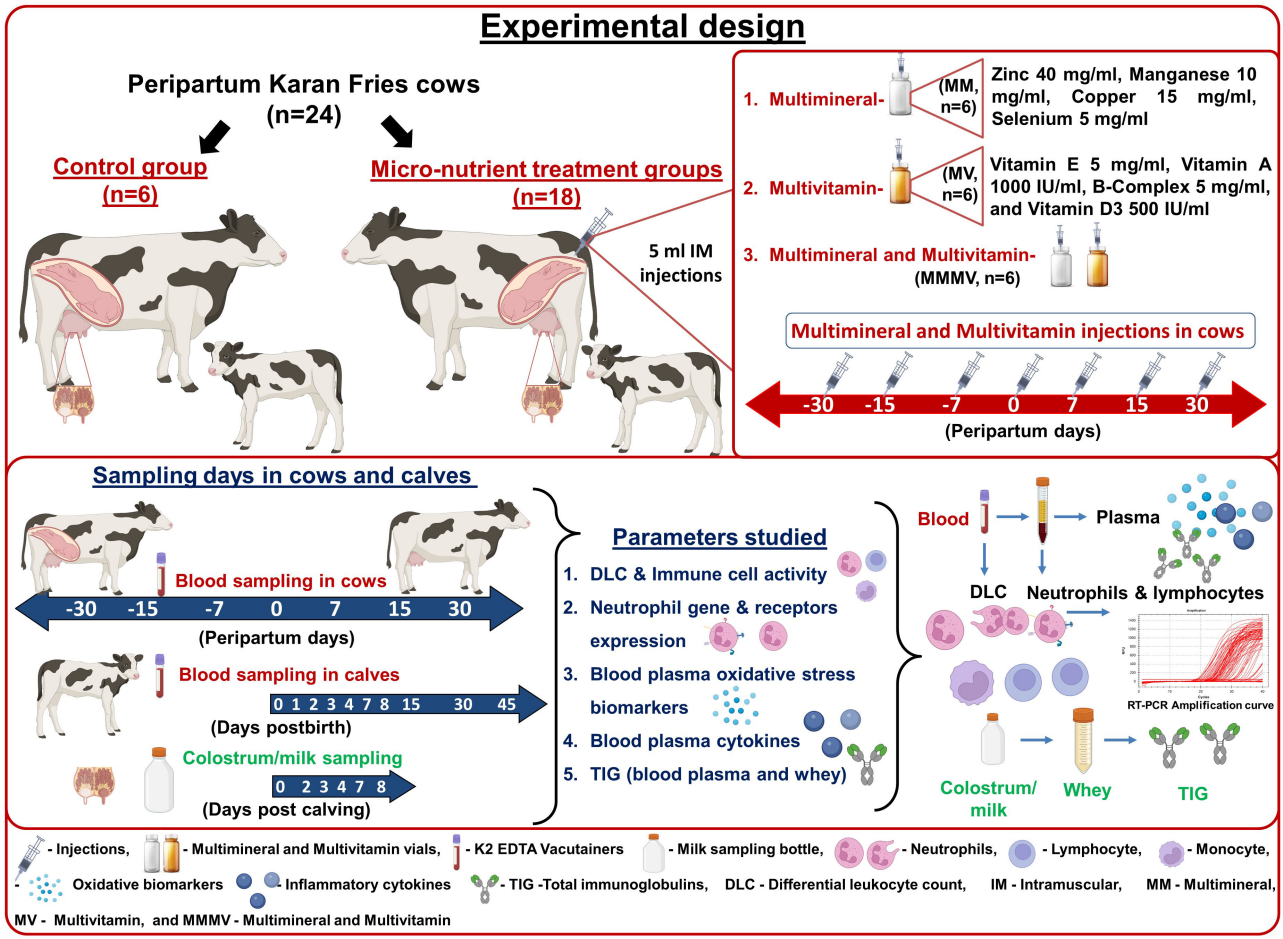
Schematic representation of the experimental design with the treatment of the transition dairy cows, the samples collected, and the parameters investigated.

Experimental cows were randomly grouped into four, having six cows in each: control, Multi-mineral (MM), Multi-vitamin (MV) and Multi-minerals and Multi-vitamin (MMMV). The control group of cows did not receive any extra mineral or vitamin injections. However, the treatment groups were injected with extra micro-nutrient formulations intramuscularly (IM). Five ml of the multi-mineral formulation was injected IM to the MM group, which consisted of Zinc 40 mg/ml, Manganese 10 mg/ml, Copper 15 mg/ml, Selenium 5 mg/ml (Stimvet chelated multimineral injection, Wellcon animal health, India). Five ml of multi-vitamin formulation was injected IM to MV group of cows, which consisted of Vitamin E 5 mg/ml, Vitamin A 1000 IU/ml, B-Complex 5 mg/ml, and Vitamin D_3_ 500 IU/ml (Zenex Ah HIVIT Plus, multivitamin injection, India). Animals of the MMMV group were injected IM with both (5 ml of multi-minerals and 5 ml of multi-vitamins) consisting of the same formulation (30–33, 35). In all the treatment groups, injections were administered on the 30^th^, 15^th^, 7^th^ days before the expected date of parturition, on the day of parturition and on days 7^th^, 15^th^ and 30^th^ after parturition (**Figure 1**).

### 2.4 Sampling schedule and blood plasma and whey separation

The calving date was predicted as ± one week before or after the projected date of calving. Accordingly, sampling started one week before the expected dates taken in the schedule. However, after calving, collected samples were regrouped according to the calving date, and only the desired samples were kept for analysis. Blood samples were collected from the cows (10 ml) before injection of micronutrients on 30^th^, 15^th^, 7^th^ days before the expected date of parturition, on the day of parturition and on days 7^th^, 15^th^ and 30^th^ after parturition and in calves, 6 ml of blood were collected from each newborn calves at birth i.e., day 0 and on days 1, 2, 3, 4, 7, 8, 15, 30 and 45 post-calving *via* jugular vein puncture in sterile K2 EDTA vacutainer tubes (Greiner bio-one, Vacuette, Austria) for neutrophils, lymphocytes isolation and plasma separation. For the estimation of total Ig (TIG) in the blood of the newborn calves, one blood sample was collected at 24 hrs after first colostrum feeding. The blood samples were brought to the lab in an ice box as soon as they were collected for further procedures.

Each cow’s colostrum and milk samples representing all four quarters were taken in sterile tubes (200 ml/cow) on the day of calving (day 0) and at days 2, 4, and 8 post-calving. Colostrum and milk samples were then centrifuged at 4000 g for 30 minutes at 4°C. The top layer containing fat was removed and rennet (0.25 µg/ml, Sigma, Missouri, USA) was added to the defatted colostrum to precipitate casein. Samples were mixed gently several times and incubated at 37°C for 30 minutes. Thereafter the samples were centrifuged at 3000 g for 10 minutes and supernatant or whey was separated and stored in 10 ml tubes at -20°C for further use.

### 2.5 Differential leukocyte count (DLC)

To determine the percentage of different immune cells, such as neutrophils, lymphocytes, and monocytes, differential leukocyte counting (DLC) was carried out. A blood smear was prepared and stained with May-Grunwald stain and Giemsa stain (Sigma-Aldrich, St. Louis, MO, USA) for (1, 2) min, respectively. A 100x under an Olympus Ix51 microscope (Olympus, Tokyo, Japan) was used to view the stained blood smear. Segmented neutrophils had a multilobed nucleus, while band neutrophils were distinguished by having a curved nucleus that was not lobar in shape.

### 2.6 Isolation of blood neutrophils and lymphocyte

Histopaque 1119 and 1077 (Sigma Aldrich, St. Louis, MO, USA) were used to separate blood neutrophils and lymphocytes from cows and calf samples using the gradient density centrifugation technique as reported by Sheikh et al. (36) and Mohapatra et al. (37). In a 15 ml polypropylene falcon tube, 3 ml of Histopaque 1119 solution was added to the tube’s bottom and 3 ml of Histopaque 1077 solution was carefully layered on top of it. An equal amount of whole blood i.e., 6 ml was taken and gradually layered over the Histopaque 1077 solution gently through the wall of the tube. Centrifugation was carried out at 900 × g for 40 min at room temperature (RT). Lymphocytes were separated as a layer of ring just above the Histopaque 1077 solution and neutrophils at the interface of the Histopaque 1119 and Histopaque 1077 layers. Neutrophils (3 ml) and lymphocytes (3 ml) were carefully transferred to new 15 ml falcon tubes separately. To remove any remaining red blood cells (RBC), neutrophils and lymphocytes pellets were slowly mixed with an equal volume of RBC lysis buffer (HiMedia Laboratories, Pennsylvania, USA). The tube was left undisturbed for 5 minutes, then centrifuged at 1000 g for 10 minutes at RT and the supernatant was discarded. The collected neutrophils and lymphocytes pellets were washed with cold Dulbecco Phosphate-Buffered Saline (PBS, Himedia, India Pvt. Ltd.) at 300 × g for 10 min at 4°C and suspended in Roswell Park Memorial Institute (RPMI) media (Catalog No. R8758, Sigma Aldrich, St. Louis, MO, USA) containing phenol red, L-glutamine and sodium bicarbonate and supplemented with 10% FBS (Catalog No SH3039602 Cytiva HyClone™, Thermoscientific, Canada), 250 μg/ml amphotericin B (Catalog No. A2942, Sigma Aldrich, St. Louis, MO, USA), 100 μg/ml streptomycin sulphate (15140-148, Life Technologies, NY, USA) and 100 U/ml penicillin G (15140-148, Life Technologies, NY, USA). Haemocytometer (Reinfeld, Germany) was used to determine the number and viability of neutrophils and lymphocytes using the 0.4% Trypan blue (Sigma, St. Louis, MO, USA) method. The viability of neutrophils and lymphocytes in the blood was greater than 96% in the first hours after neutrophil processing and gradually decreased thereafter. Using the May-Grunwald-Giemsa staining method mentioned above, the purity of the neutrophils and lymphocytes population was determined and was greater than 95%.

### 2.7 *In vitro* phagocytic activity (PA) of blood neutrophils and proliferation assay of lymphocyte

*In vitro* PA of the isolated blood neutrophils from cows and calves was estimated by calorimetric method using Nitro blue tetrazolium (NBT) assay as described by Sim Choi et al. (38), Dang et al. (21) and Sheikh et al. (36). Approximately 5x10^6^ neutrophils/ml were added to a 96-well tissue culture plate (Coster, Sigma Aldrich USA) using RPMI-1640 media containing phenol red, L-glutamine and sodium bicarbonate and supplemented with 10% FBS, 250 μg/ml amphotericin B, 100 μg/ml streptomycin sulphate and 100 U/ml penicillin G. The cells were allowed to proliferate with 650 μg/ml of zymosan-A (Catalog No. Z4250, Sigma Aldrich, St. Louis, MO, USA) and 250 μg/ml of NBT (Catalog No. N6876, Sigma Aldrich, St. Louis, MO USA). As a negative control, neutrophils were cultured with NBT and culture media without zymosan. The cultures were subsequently incubated for 3 hours in a humidified CO_2_ incubator (37°C, 95% air and 5% CO_2_). The amount of zymosan phagocytosed was used as an indicator of PA. Nitro blue tetrazolium was used to determine the production of superoxide anion ( ${\text{O}}_{2}^{-}$ ) in the neutrophils. Nitro blue tetrazolium is yellow in colour but is changed to blue formazan after phagocytosis. Finally, the optical density (OD) was measured at 540 nm by ELISA reader (MultiSkan GO, Thermo Scientific, Finland).

The lymphocyte proliferation assay was estimated using the colorimetric MTT (Methyl thiazolyldiphenyl-tetrazolium bromide, MP Biomedicals, India) assay as described by Dang et al. (21) with minor modifications. Briefly, the cell suspension of lymphocytes was adjusted to 5×10^6^ live cells/ml by the RPMI culture media containing phenol red, L-glutamine and sodium bicarbonate and supplemented with 10% FBS, 250 μg/ml amphotericin B, 100 μg/ml streptomycin sulphate and 100 U/ml penicillin G. The mitogen employed in this study was concanavalin A (50 µg/ml), which stimulated lymphocytes. The cells were allowed to proliferate with and without mitogen to determine the difference between cell proliferations. All cultures were incubated at 37 °C in a humidified CO_2_ incubator (95% air and 5% CO_2_) for 72 h. After adding MTT dye and incubating it for 4 hours, formazan crystals were formed. To dissolve the dark blue formazan crystals, 100 µl of DMSO (Dimethyl sulphoxide) was then added to each well and thoroughly mixed. The OD was measured using an ELISA reader (MultiSkan GO, Thermo Scientific, Finland) in a dual wavelength-measuring system at a test wavelength of 503 nm and a reference wavelength of 630 nm after the plate had been incubated at room temperature for 15 min.

### 2.8 RNA isolation from blood neutrophils, cDNA synthesis and real-time polymerase chain reaction (RT-PCR)

To conduct expression studies, neutrophils isolated from cows and calves were adjusted to 1x10^6^ live cells/ml. According to the manufacturer’s instructions, total RNA from blood neutrophils was extracted and purified using the TRIzol reagent (Invitrogen, Carlsbad, CA). RNase-Free DNase Set (Qiagen, India Pvt. Ltd.) was used to remove the genomic DNA contamination. The integrity of the RNA was evaluated by agarose gel electrophoresis (1.5% agarose), and the quantity and quality of RNA were verified by OD absorption ratio at λ_260_/λ_280_ using Bio Spec-nano (serial no., A116449; Biotech). A ratio of 1.9 to 2.0 was accepted as “pure” for RNA.

Gradient PCR was used to optimize the annealing temperature of each primer (including endogenous genes β-actin and GAPDH primer), followed by agarose gel electrophoresis (1.5%) to visualize the product size of PCR amplified products of target genes. Primers for specific bovine toll-like receptors (TLR2, TLR4), chemokine receptors (CXCR1, CXCR2), glucocorticoid receptor (GR-α), cluster of designation (CD62L, CD11b, CD25 and CD44) and endogenous genes (GAPDH, β-actin) were selected from the published literature (39–41) and shown in **Table 2** (Sigma Chemicals Co., St. Louis, Missouri, USA). Quantitative real-time PCR (qPCR) (Roche’s Lightcycler 480) was carried out using Thermo Scientific Maxima SYBR Green qPCR Master Mix kit (Thermo Scientific, USA) in accordance with the manufacturer’s instructions. Briefly, template cDNA (1 μl), SYBR green (2x) mixes (5 μl), forward and reverse primers (0.5 μl each) and total reaction volume up to 10 μl was made using nuclease-free water. The protocol of RT-PCR consisted of 45 cycles at 95°C for 15 s, annealing at 58 or 59°C for 20 s, and performed denaturation kinetics to assess the reaction product. β-actin and GAPDH were used as endogenous genes and the mRNA abundance of the day -30 for cows and day of birth for calves was taken as a calibrator with which relative expression of all genes during different time points was estimated. The relative quantification of all genes was evaluated by the 2^−ΔΔCt^ method (42).

**Table 2:** Details of various primers used in the study.

Genes	Sequence (5′→3′)	Accession. No.	Size (bp)	Annealing Temp (°C)
**TLR2**	F: CTGGCAAGTGGATTATCGACAA R: TACTTGCACCACTCGCTCTTCA	XM_005217446.4	103	59
**TLR4**	F:TGCGTACAGGTTGTTCCTAACATT R:TAGTTAAAGCTCAGGTCCAGCATCT	NM_174198.6	110	59
**CXCR1**	F: AGTCCCCGTGAGATAAGCAC R: CCAGGTTCAGCAGGTAGACA	EF597244.2	163	59
**CXCR2**	F: CAACACTGA-CCTGCCCTCTA R: CCAGGTTCAGCAGGTAGACA	DQ328664.1	197	59
**GRα**	F: TGTGGTTTAAAGAGGGCCAAG R: TTCTACGTTCCCATCACTGAAAAG	XM_024993840.1	74	58
**CD62L**	F: CCGATTGCTGGACTTACCAT R: CCAAGTCCACACCCCTTCTA	NM_174182.1	194	58
**CD11b**	F: TAAGAAGAGCCCGGTGCTGAAC R: TGGGATGGCACACTGGATTCTC	NM_001039957.1	63	59
**CD25**	F: ACATCGGCAGTGGTCTCAG R: GAACCTCCACATCAGCAAGC	NM_174358.2	60	58
**CD44**	F: CTGTCAACAGTAGGAGAAGGTGTG R: TCCTCCATGGTTCCATTCCCATTG	NM_174013.3	73	58
**GAPDH**	F: GGGTCATCATCTCTGCACCT R: GGTCATAAGTCCCTCCACGA	NM_001034034	176	59
**β-actin**	F: ACAGTCCGCCTAGAAGCA R: TGGCACCCAGCACAATGAAGATC	BT030480.1	179	58

F, Forward; R, Reverse; TLR2 and TLR4, Toll like receptors; CXCR1 and CXCR2, Chemokine receptors; GRα, Glucocorticoid receptor; CD62L, CD11b, CD25 and CD44, Cluster of designation molecules; GAPDH and β-actin, housekeeping genes.

### 2.9 Quantification of oxidative stress biomarkers

QuantiChrom™ assay kits (Bioassay systems, Hayward, CA, USA) were used for estimating the concentration of blood plasma thiobarbituric acid reactive substances (TBARS) and total antioxidant capacity (TAC) (43, 44). The detectable limit for TBARS (DTBA-100) was 1 - 30 nmol/ml and for TAC was 0.0015 to 1 mmol/l (DTAC-100). The intra-assay and inter-assay variations were less than 8% and 10%, respectively. Furthermore, bovine-specific ELISA kits were used to determine the blood plasma enzymatic activity of superoxide dismutase (SOD, Wuhan Fine Biotech, Wuhan, China) and catalase (CAT, Bioassay Technology Laboratory, Shanghai, China). The sensitivity of SOD (Catalogue No.- EB0164) and CAT (Cat No.- E0025Bo) were 0.469 ng/ml and 0.28 ng/ml, respectively (45). The standard curve range was 0.781-50 ng/ml for SOD and 0.5-200 ng/ml for CAT. The intra-assay and inter-assay variations were less than 8% and 10%, respectively, for both SOD and CAT. The optical density (OD) was measured by an ELISA reader (Multiskan Go, Thermo Scientific, Finland).

### 2.10 Quantification of plasma cytokines

Pro-inflammatory (IL-1α, IL-1β, IL-6, IL-8, IL-17A, IFN-γ and TNF-α) and anti-inflammatory cytokines (IL-4 and IL-10) were analyzed simultaneously using MILLIPLEX^®^ Bovine Cytokine/Chemokine Magnetic Bead Panel 1 - Immunology Multiplex Assay kit (Cat. # BCYT1-33K, Merck life sciences, Darmstadt, Germany) based on the Luminex^®^ xMAP^®^ technology. Details of assay characteristics for all cytokines in the Multiplex assay kit are mentioned in **Table 3**.

**Table 3:** Assay characteristics for all cytokines of MILLIPLEX^®^ bovine cytokine immunology multiplex assay (Cat. # BCYT1-33K).

Cytokine	Accuracy	Sensitivity	Standard Curve Range	Intra-assay %CV	Inter- assay %CV
**IL-1α**	94%	0.36	0.3 - 5,000 pg/ml	<10%	<10%
**IL-1β**	91%	4.93	2.6 - 40,000 pg/ml	<10%	<10%
**IL-6**	95%	11.23	2.6 - 40,000 pg/ml	<10%	<15%
**IL-8**	95%	22.6	2.2 - 35,000 pg/ml	<10%	<10%
**IL-17A**	103%	0.67	0.6 - 10,000 pg/ml	<10%	<10%
**IFN-γ**	94%	0.08	0.1 - 2,000 pg/ml	<10%	<10%
**TNF-α**	92%	22.62	12.8 - 200,000 pg/ml	<10%	<15%
**IL-4**	93%	16.57	12.8 - 200,000 pg/ml	<10%	<10%
**IL-10**	96%	1.05	0.96 - 15,000 pg/ml	<10%	<10%

IL, Interleukin; IFN-γ, Interferon gamma; TNF-α, Tumor necrosis factor.

### 2.11 Quantification of TIG by competitive ELISA

Bovine-specific ELISA kit (Southern California, San Diego, USA) was used to determine the total immunoglobulins (TIG) concentration in the whey of cows’ colostrum/milk and calf’s blood samples, respectively. The colostrum samples were diluted with PBS in ratio 1:2. The minimum detectable limit for Total Immunoglobulins (MyBioSource. Cat No. - MBS5738656) was 1.0 mg/ml. The accuracy of the assay is 92-101% and the maximum detection value is 100 mg/ml. The intra-assay and inter-assay deviations were less than 10%. The optical density (OD) was measured by an ELISA reader (Multiskan Go, Thermo Scientific, Finland).

### 2.12 Statistical analysis

The data was analyzed by using the SPSS software system (version 22). All the data obtained from the study were expressed as mean ± standard error and were analyzed by two-way ANOVA. Tukey"s multiple comparison test was used to find significance at the 5 percent level. The effect of treatment group (Gr), days (D) of the periparturient period, and their interactions (Gr×D) were estimated using the statistical model shown below


$$Y_{ijk}=\text{µ}+Gr_{i}+D_{j}+\left(GrD\right)ij+e_{ijk}$$

where, Y_ijk_ is a dependent variable, μ is the overall mean of the population, Gr_i_ is the effect of micronutrients feeding (i = 4), D_j_ is the effect due to the measurement days (j = 4 - 7 based on parameters studied), and (GrD)_ij_ is the effect due to treatment group by measurement days’ interactions, and e_ijk_ is the residual error.

## 3 Results

### 3.1 Blood differential leucocytes count (cow, calf)

#### 3.1.1 Percentage and type of neutrophils

The mean total percentage of neutrophils in blood and immature banded neutrophils was highest in the control group (P<0.05) and decreased significantly in response to micronutrient injection, with the lowest values recorded in MMMV injected cows and their calves, followed by MM and MV groups. The percentage of blood neutrophils and banded neutrophils was highest in cows on the day of calving and in calves on the day of birth. The viability of neutrophils and lymphocytes was decreased at the time of calving in cows and at the time of birth in calves and were comparatively higher in the MMMV group as compared to the control group (**Supplementary Tables 1**, **2**).

**Figure 2:**
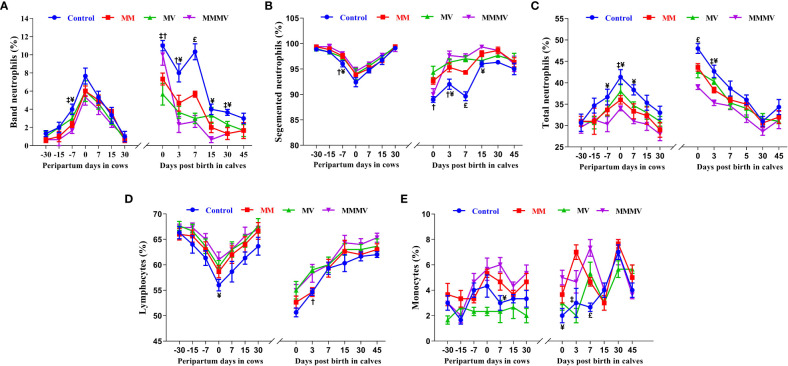
Blood differential leucocytes count including the percentage of band neutrophils **(A)**, segmented neutrophils **(B)**, total neutrophils **(C)**, lymphocytes **(D)**, and monocytes **(E)** in control and treatment groups of crossbred cattle and their calves. Control: received basal diet only, MM: basal diet + injectable multimineral, MV: basal diet + injectable multivitamins, MMMV: basal diet + combination of injectable multimineral and multivitamins. The mean values with different superscript symbols represent significant differences with control group at different days of peripartum in cows and post birth in calves; ^‡^Control Vs MM, ^†^Control Vs MV, ^¥^Control Vs MMMV. ^£^Control Vs MM, MV and MMMV.

The percentage of segmented neutrophils showed an inverse pattern compared to that of banded neutrophils, where it decreased significantly at the time of calving in cows and on the day of birth in calves. It was also significantly (P<0.05) lower in the control groups of cows and calves compared to the treatment groups, with the highest values recorded in the MMMV-injected group (**Figures 2A–C**; **Supplementary Tables 3**, **4**).

#### 3.1.2 Percentage of blood lymphocytes and monocytes

The percentage of blood lymphocytes showed an inverse pattern to that of blood neutrophils, being lowest in the control group (P<0.05) and increasing significantly in response to micronutrient injection. In cows, the percentage of blood lymphocytes decreased towards calving, with the lowest values recorded on the day of calving and starting to increase as lactation progressed (P<0.05), reaching the highest values on day 30 after calving. In calves, the lowest percentage of blood lymphocytes was observed on the 0th and 3rd day of life and increased with the following days of life (**Figure 2D**; **Supplementary Tables 3**, **4**).

In cows and calves, the percentage of blood monocytes was lowest in the MV and control groups (P<0.05) and increased significantly in the MMMV and MM groups. In cows, the percentage of blood monocytes increased until calving and started to decrease as lactation progressed (P<0.05). In the calves, the highest percentage of blood monocytes was observed on day 0 in the MMMV group, and on day 3 in the MM group (**Figures 2E**; **Supplementary Tables 3**, **4**).

#### 3.1.3 Neutrophil phagocytic activity (PA) and lymphocyte proliferation assay (LPA)

**Figure 3:**
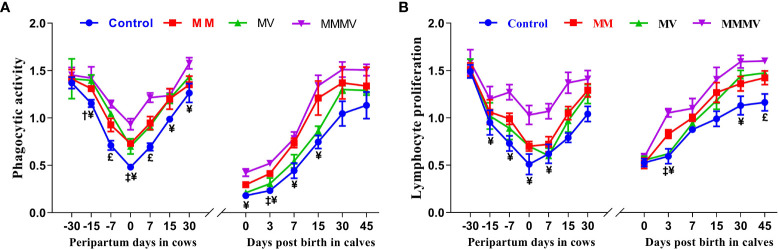
Phagocytic activity of neutrophils (PA, A) and lymphocytes proliferation (LPA, B) in control and treatment groups of crossbred cattle and their calves. Control: received basal diet only, MM: basal diet + injectable multimineral, MV: basal diet + injectable multivitamins, MMMV: basal diet + combination of injectable multimineral and multivitamins. The mean values with different superscript symbols represent significant differences with the control group at different days of peripartum in cows and post birth in calves; ^‡^Control Vs MM, ^†^Control Vs MV, ^¥^Control Vs MMMV. ^£^Control Vs MM, MV and MMMV.

In both the cow and calves, mean blood neutrophil PA and lymphocyte proliferation were lowest in the control group (P<0.05) and increased significantly in response to micronutrient injection. In addition, PA and LPA decreased towards calving, with the lowest activity measured on the day of calving and increasing as lactation progressed (P<0.05), reaching the highest values on day 30 after calving. In all calf groups, the lowest values of PA and LPA were observed on the 0th and 3rd day of life and increased with the following days of life. Although days and groups had a significant effect on PA and LPA, the interaction between days and groups was not significant in the cows and their calves during the study period (**Figures 3A, B**; **Supplementary Tables 5**, **6**).

### 3.2 Gene and receptor expression in blood neutrophils (cow, calf)

#### 3.2.1 Relative mRNA expression of toll-like, chemokine and glucocorticoid receptors in blood neutrophils

**Figure 4:**
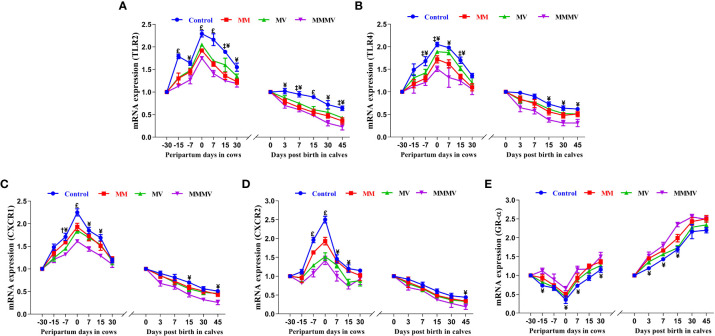
Relative mRNA expression of TLR2 **(A)**, TLR4 **(B)**, CXCR1 **(C)**, CXCR2 **(D)**, and GR-α **(E)** in blood neutrophils of control and treatment groups of crossbred cattle and their calves. Control: received basal diet only, MM: basal diet + injectable multimineral, MV: basal diet + injectable multivitamins, MMMV: basal diet + combination of injectable multimineral and multivitamins. The mean values with different superscript symbols represent significant differences with the control group at different days of peripartum in cows and post birth in calves; ^‡^Control Vs MM, ^†^Control Vs MV, ^¥^Control Vs MMMV. ^£^Control Vs MM, MV and MMMV. β-actin and GAPDH were used as endogenous genes and the mRNA abundance of the day -30 for cows and day of birth for calves was taken as a calibrator with which relative expression of all genes during different time points was estimated.

The relative mRNA expression of toll-like receptors (TLR2 and TLR4) and chemokine receptors (CXCR1 and CXCR2) in blood neutrophils showed a similar pattern in both cows and their calves throughout the study period. In both cows and calves, the average relative expression of TLRs and CXCRs was higher (P<0.05) in the control group and decreased in response to micronutrient injection, with the lowest expression recorded in the combination injection group, followed by the MM and MV groups. In all cow groups, the relative expression of TLRs was lowest one month before calving, increased as calving approached, with the highest expression recorded on the day of calving, and then decreased to pre-calving levels. In calves, the highest relative mRNA expression of TLRs and CXCRs was observed on the day of birth, while the lowest expression was recorded on the 30th and 45th day of life. The relative mRNA expression of the glucocorticoid receptor (GR-α) in blood neutrophils showed an inverse pattern to that of TLRs and CXCRs throughout the study period. Different treatment groups and sampling days had significant (P ≤ 0.001) effects on the expression of TLRs, CXCRs and GR-α in both cows and their calves (**Figures 4A–E**; **Supplementary Tables 7**, **8**).

#### 3.2.2 Relative mRNA expression of cluster of designation molecules in blood neutrophils

**Figure 5:**
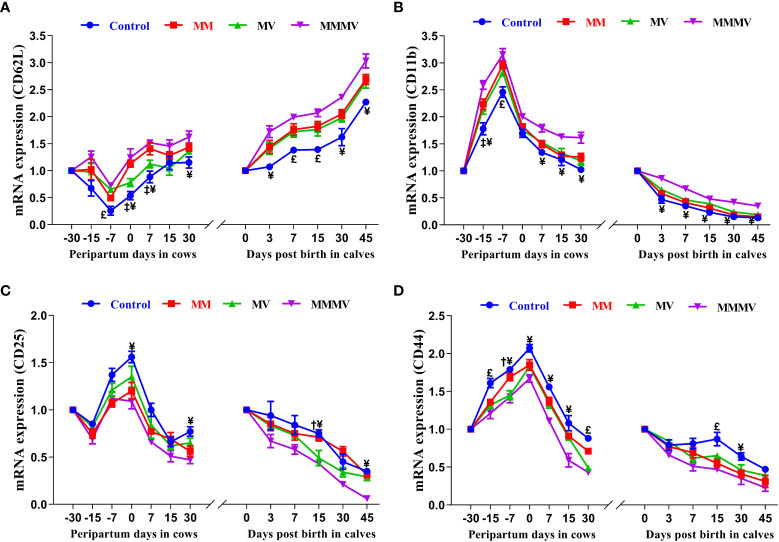
Relative mRNA expression of CD62L **(A)**, CD11b **(B)**, CD25 **(C)**, and CD44 **(D)** in blood neutrophils of control and treatment groups of crossbred cattle and their calves. Control: received basal diet only, MM: basal diet + injectable multimineral, MV: basal diet + injectable multivitamins, MMMV: basal diet + combination of injectable multimineral and multivitamins. The mean values with different superscript symbols represent significant differences with the control group at different days of peripartum in cows and post birth in calves; ^‡^Control Vs MM, ^†^Control Vs MV, ^¥^Control Vs MMMV. ^£^Control Vs MM, MV and MMMV. β-actin and GAPDH were used as endogenous genes and the mRNA abundance of the day -30 for cows and day of birth for calves was taken as a calibrator with which relative expression of all genes during different time points was estimated.

The relative mRNA expressions of CD11b, CD25 and CD44 in blood neutrophils showed a similar trend in both the cow and calf groups. In all cow groups, the relative expression of C11b, CD25 and CD44 increased as calving approached, with the highest values recorded on the day of calving. Thereafter, the expression of CD25 and CD44 began to decrease as lactation progressed. In contrast to CD25 and CD44, the relative expression of CD11b remained constant in the second week of lactation and increased again on day 30 after calving in all groups. In calves, the relative expression of CD11b, CD25 and CD44 was highest on the day of birth and decreased in all groups in the following weeks of life. The relative expression of CD62L showed an inverse pattern compared to the other CDs, being lowest on the day of calving in cows and on the day of birth in calves. In both cows and calves, the mean relative overall expression of CD25 and CD44 in blood neutrophils was higher in the control group and decreased in the treated groups (P<0.05), with the lowest expression recorded in the MMMV group, followed by the MV and MM groups. In contrast to CD25 and CD44, the mean relative overall expression of CD62L and CD11b in blood neutrophils was higher in both cows and calves in the MMMV group (P<0.05) than in the control group. Different treatment groups and sampling days had (P<0.05) effects on the expression of all the cluster of designation molecules in both cows and their calves. However, groups by days interactions have significant (P<0.05) effects on the expression of CD44 in cows and calves and on the expression of CD11b in cows (**Figures 5A–D**; **Supplementary Tables 9**, **10**).

### 3.3 Concentrations of biomarkers for oxidative stress in blood plasma (cow, calf)

**Figure 6:**
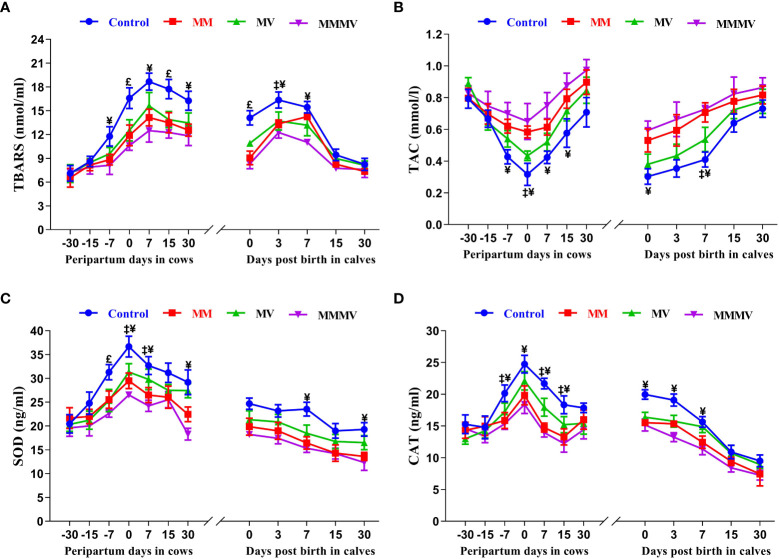
The blood plasma concentration of oxidative stress biomarkers including thiobarbituric acid reactive substances (TBARS, nmol/ml), total antioxidant capacity (TAC, mmol/l), superoxide dismutase (SOD, ng/ml), and catalase (CAT, ng/ml) in control and treatment groups of crossbred cattle and their calves (**A-D**), respectively. Control: received basal diet only, MM: basal diet + injectable multimineral, MV: basal diet + injectable multivitamins, MMMV: basal diet + combination of injectable multimineral and multivitamins. The mean values with different superscript symbols represent significant differences with the control group at different days of peripartum in cows and post birth in calves; ^‡^Control Vs MM, ^†^Control Vs MV, ^¥^Control Vs MMMV. ^£^Control Vs MM, MV and MMMV.

Oxidative stress biomarker concentrations showed a similar trend in both the cow and calf groups. In all cow groups, TBARS concentrations increased as calving approached, with the highest values recorded one week after calving. Thereafter, TBARS concentrations began to decrease as lactation progressed. In contrast, TAC showed an inverse pattern compared to other oxidative stress biomarkers, being lowest in cows on the day of calving and in calves on the day of birth. SOD and CAT activity showed a comparable profile, increasing towards calving days, with the highest activity measured on the day of calving and decreasing as lactation progressed (P<0.05), with the lowest values recorded on day 30 after calving. In the calves, TBARS concentration, SOD and CAT activity were highest on the day of birth and decreased in all groups during the following weeks of life. In both cows and calves, mean TBARS concentration, SOD and CAT activity were highest in the control group (P<0.05) and decreased significantly in response to micronutrient injection. Overall, TAC was higher (P<0.05) in both cows and calves in the MMMV group than in the control group. In addition, a significant difference in TBARS between the control and MMMV groups was observed on days 0, 7 and 15 of calving. TAC increased significantly in response to MMMV injections on days 7, 0 and 7 of calving, while SOD and CAT levels decreased. Different treatment groups and sampling days had significant effects (P< 0.001) on the concentration of all oxidative stress biomarkers in both cows and their calves (**Figures 6A–D**; **Supplementary Tables 11**, **12**).

### 3.4 Blood inflammatory cytokines concentrations (cow, calf)

#### 3.4.1 Pro-inflammatory cytokines

**Figure 7:**
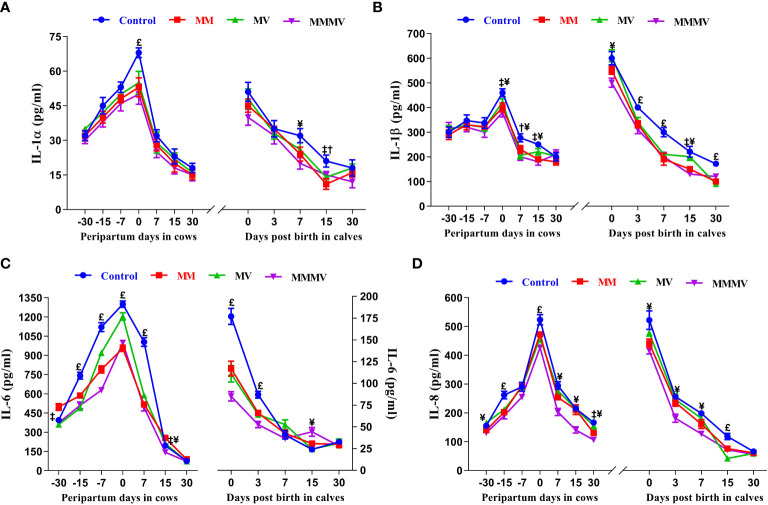
The blood plasma concentration of pro-inflammatory cytokines (pg/ml) including IL-1α **(A)**, IL-1β **(B)**, IL-6 **(C)**, and IL-8 **(D)** in control and treatment groups of crossbred cattle and their calves. Control: received basal diet only, MM: basal diet + injectable multimineral, MV: basal diet + injectable multivitamins, MMMV: basal diet + combination of injectable multimineral and multivitamins. The mean values with different superscript symbols represent significant differences with the control group at different days of peripartum in cows and post birth in calves; ^‡^Control Vs MM, ^†^Control Vs MV, ^¥^Control Vs MMMV. ^£^Control Vs MM, MV and MMMV.

**Figure 8:**
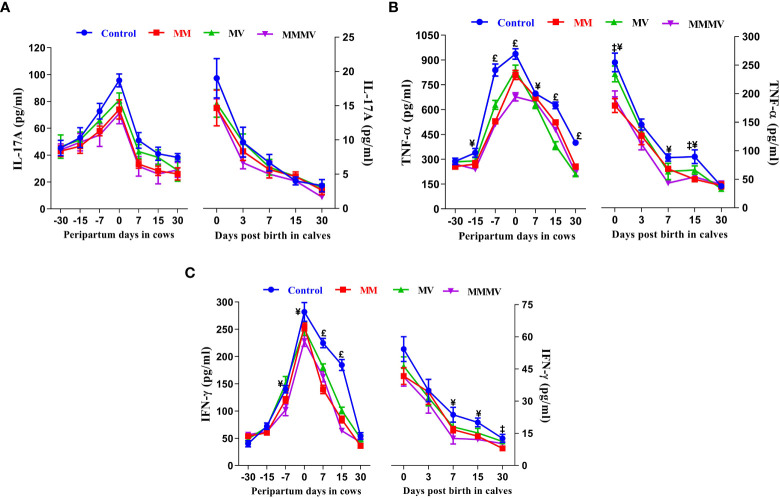
The blood plasma concentration of pro-inflammatory cytokines (pg/ml) including IL-17A **(A)**, TNF - α **(B)**, and IFN-γ **(C)** in control and treatment groups of crossbred cattle and their calves. Control: received basal diet only, MM: basal diet + injectable multimineral, MV: basal diet + injectable multivitamins, MMMV: basal diet + combination of injectable multimineral and multivitamins. The mean values with different superscript symbols represent significant differences with the control group at different days of peripartum in cows and post birth in calves; ^‡^Control Vs MM, ^†^Control Vs MV, ^¥^Control Vs MMMV. ^£^Control Vs MM, MV and MMMV.

The concentration of all pro-inflammatory cytokines in both the cows and their calves showed a similar pattern throughout the study period. In both cows and calves, the overall mean levels of pro-inflammatory cytokines were significantly higher in the control group and decreased in response to micronutrient injection, with the lowest concentration recorded in the combination injection group, followed by the MM and MV groups. In cows, the concentration of IL-1α, IL-1β and IL-17A remained unchanged until the day of calving. In contrast, the concentration of IFN-γ and TNF-α remained unchanged until one week before calving. However, the concentration of IL-6 and IL-8 increased significantly from one month before calving. Further, the concentration of all pro-inflammatory cytokines started to increase as calving approached and attained the highest values on the day of calving. Subsequently, the concentration of all pro-inflammatory cytokines started decreasing (P<0.05) with the progress of lactation and attained the lowest values at day 30 after calving in all the groups. In calves, a significantly higher concentration of all pro-inflammatory was observed in all groups of calves on day 0 and day 3, which then decreased on the following days (i.e., 7, 15 and 30 days). The days of sampling and treatment had significant (P ≤ 0.001) effects on the concentration of all the pro-inflammatory cytokines in both cows and their calves. However, the treatment had no impact on the concentration of IL-1α, IL-17A and IFN-γ in calves. Groups by days interactions had significant (P ≤ 0.001) effects on the concentration of IL-6 in cows and their calves and on the concentration of IFN-γ in cows (**Figures 7A–D**, **8A–C**; **Supplementary Tables 13** **-** **16**).

#### 3.4.2 Anti-inflammatory cytokines

**Figure 9:**
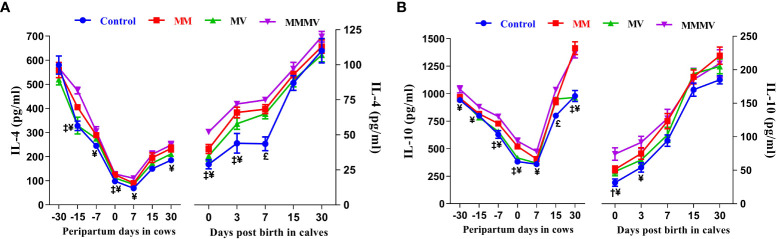
The blood plasma concentration of anti-inflammatory cytokines (pg/ml) including IL-4 **(A)**, and IL-10 **(B)** in control and treatment groups of crossbred cattle and their calves. Control: received basal diet only, MM: basal diet + injectable multimineral, MV: basal diet + injectable multivitamins, MMMV: basal diet + combination of injectable multimineral and multivitamins. The mean values with different superscript symbols represent significant differences with the control group at different days of peripartum in cows and post birth in calves; ^‡^Control Vs MM, ^†^Control Vs MV, ^¥^Control Vs MMMV. ^£^Control Vs MM, MV and MMMV.

Throughout the study period, the concentrations of IL-4 and IL-10 followed a similar trend in both cows and their calves. In cows, the concentration of IL-4 and IL-10 was highest one month before calving and decreased in the following weeks. The concentration of IL-4 reached the lowest values one week after calving. The concentration of IL-10, on the other hand, reached the lowest values on the day of calving. Thereafter, the IL-4 and IL-10 concentrations gradually increased as lactation progressed and reached the highest values in all groups on day 30 after calving. In the calves, significantly lower levels of IL-4 and IL-10 were observed in all groups on day 0 and day 3, which then increased on the following days (i.e., 7, 15 and 30 days). In both cows and calves, the overall mean levels of IL-4 and IL-10 were significantly lower in the control group. These levels increased in response to micronutrient injection, being highest in the MMMV group, followed by MM and MV. However, IL-10 levels in the MV groups did not differ (P<0.05) from those in the control group. In addition, IL-4 levels (P<0.05) increased in response to MMMV injections on the 0th, 3rd and 7th day of life of calves compared to control. In contrast, IL-10 levels increased (P<0.05) in response to MMMV injections in calves on day 0 and day 3. Different days and treatment groups had significant (P ≤ 0.001) effects on the levels of IL-4 and IL-10 in both cows and their calves. In addition, interactions between days and groups had significant (P<0.05) effects on the concentration of IL-10 in cows (**Figures 9A, B**; **Supplementary Tables 17**, **18**).

### 3.5 Total Immunoglobulin (TIG) levels (cow, calf)

**Figure 10:**
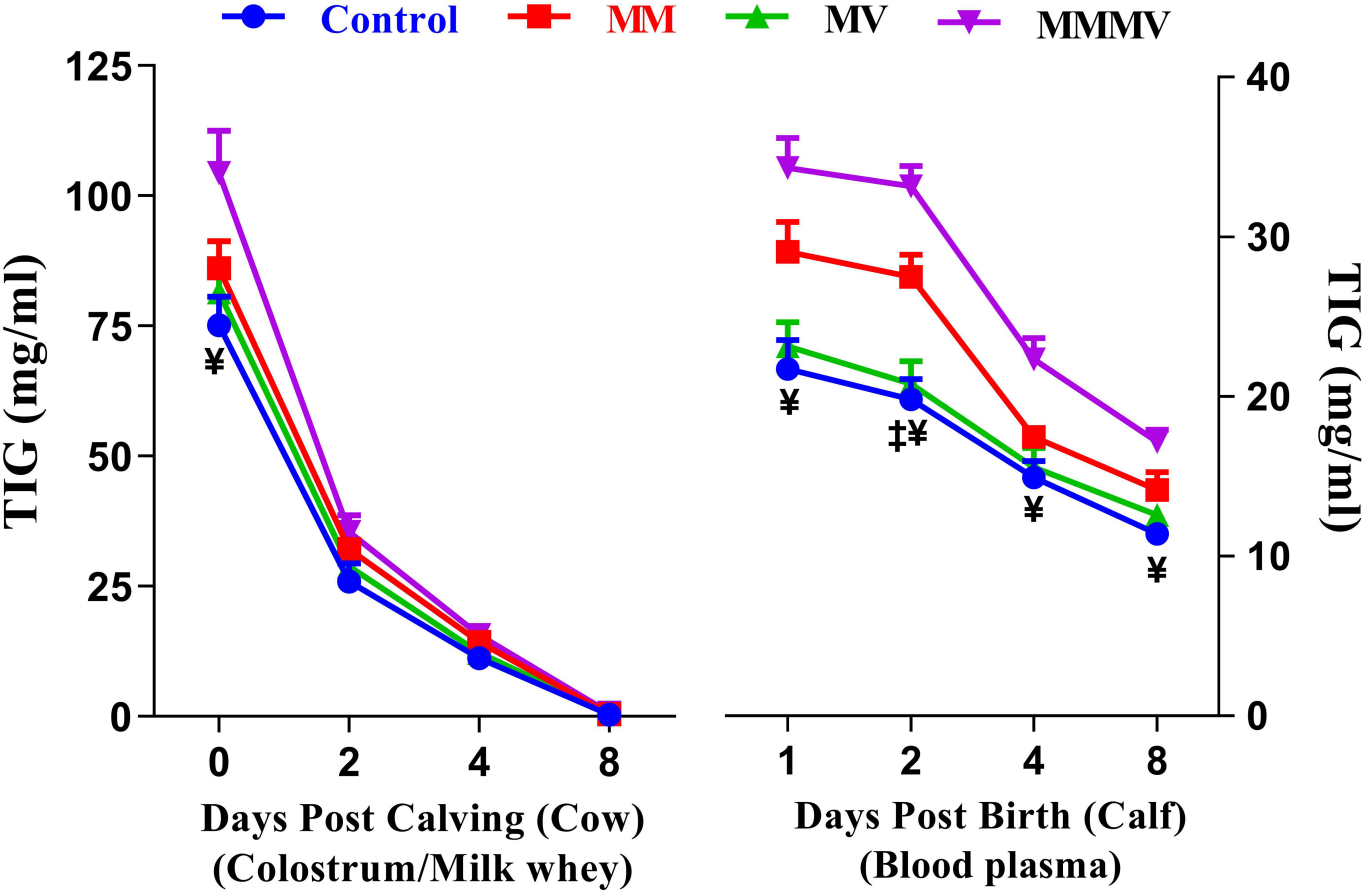
Total immunoglobulins (TIG) concentration (mg/ml) in colostrum/whey of different groups of Karan-Fries cows and TIG concentration (mg/ml) in the blood plasma of the calves born to different groups of Karan-Fries cows. Control: received basal diet only, MM: basal diet + injectable multimineral, MV: basal diet + injectable multivitamins, MMMV: basal diet + combination of injectable multimineral and multivitamins. The mean values with different superscript symbols represent significant differences with the control group at different days of peripartum in cows and post birth in calves; ^‡^Control Vs MM, and ^¥^Control Vs MMMV.

Different days of sampling and treatment groups had significant effects on TIG concentrations in both cows and calves. In colostrum/milk of cows, TIG concentrations reached the highest values on the day of calving and decreased (P<0.05) by more than 40-fold in the following days of lactation in all groups, with the lowest values recorded on days 4 and 8 after calving. The TIG concentration on the day of calving was significantly higher in MMMV than in the control group. In addition, the mean TIG concentration in colostrum/milk was significantly higher in the MMMV-treated groups than in the control group (P<0.05). However, the TIG concentrations in the MM and MV groups did not differ from those in the control group. In the calves, plasma TIG concentrations were highest on the day of birth in all groups and decreased significantly on the 4th and 8th day after birth (P<0.05). Plasma TIG concentrations were significantly higher in MMMV on the day of birth and on the 2nd day of life. The highest plasma TIG concentrations were observed in the calves of the MMMV and MM groups, while the lowest TIG concentrations were found in the control group of calves (P<0.05). However, TIG levels in the MV groups did not differ from those in the control group (**Figure 10**; **Supplementary Tables 19**, **20**).

## 4 Discussion

During the transition period, there is a decrease in blood levels of calcium, zinc, magnesium, phosphorous, potassium, selenium, and vitamins A and E which significantly impair many essential functions of the host defense system and predispose periparturient animals to oxidative stress, tissue damage and many health complications (15, 46). It is well documented that the decrease in the blood concentration of trace elements and vitamins during the periparturient period can impair many immune system functions and lead to various inflammatory diseases in bovines (16–19). Therefore, ensuring optimal availability of essential antioxidant micronutrients is a key and promising approach to improve the health and production of transition dairy cows and minimize the incidence of metabolic and oxidative stress-related health disorders. Indeed, in response to repeated injections of trace elements and vitamins in periparturient cows, we observed improved blood immune cell function and a decrease in inflammation and oxidative stress in transition dairy cows and their calves.

Of the leukocytes, neutrophils and lymphocytes are the major immune cells, contributing to both innate and acquired immune responses, fighting infections, and safeguarding against various diseases. Neutrophil counts peak on the day of parturition as cows are under nutritional, endocrine, and physiological stress (21, 47). In general, a lower percentage of lymphocytes on the day of calving could be related to systemic immunosuppression, which can lead to the occurrence of postpartum inflammatory diseases after birth (48, 49). In the present study, a lower percentage of total neutrophils, immature neutrophil and a higher percentage of lymphocytes were found in the micronutrient-injected groups, reflecting a lower inflammatory response and stress level, and reduced signals to the bone marrow to release immune cells compared to the control group. Neutrophil phagocytic activity and lymphocyte proliferation are primitive defense mechanisms that can be used as novel indicators of immunity and play a major role in disease control (8, 21, 40). In the current study, the week before and after calving is marked by a decrease in the phagocytic activity of neutrophils and the proliferation capacity of lymphocytes. Leukocyte function is thought to be impaired around calving due to a lack of energy, vitamin and mineral deficiencies, and an increase in oestradiol and glucocorticoids (7, 50). Although we observed a greater increase in the number of neutrophils, especially banded cells, in the calves at birth, their phagocytic activity was low compared to subsequent weeks of life, which is consistent with previous studies (51, 52). Furthermore, phagocytic activity and lymphocyte proliferation of calves in the MMMV group were significantly higher than those in the control group, which could be attributed to the improved colostrum quality, successful passive transfer of immunity and optimal availability of essential nutrients that boost immune functions. Recently, we have shown that oral administration of combined micronutrients such as vitamin A (10^5^ IU), vitamin E (2500 IU) and zinc sulphate (60 ppm) from one month before the expected calving date until calving lowers stress levels and improves the immune response of dairy cows and subsequently the well-being of their calves (12). Studies in dairy cattle have revealed that multivitamins influence the proliferation, differentiation, and function of lymphocytes and the phagocytic activity of neutrophils during stressful periods (21, 31, 53). As shown in the current study, adequate micronutrient injections during this crucial period have positive effects on immune cell activity and thus help in preventing or minimizing the incidence of postpartum diseases.

Oxidative stress occurs when the production of reactive oxygen species (ROS) exceeds the body’s antioxidant capacity and leads to suppression and impairment of proper immune system function (3). Periparturient dairy cows and neonatal calves experience high oxidative stress, which predispose them to metabolic and infectious diseases (3, 13). In the present study, total antioxidant capacity, was higher in the treated groups, while the activity of antioxidant enzymes including SOD and CAT as well as TBARS levels, a by-product of lipid peroxidation that reflects the severity of oxidative stress, were lower. Immune cells are sensitive to oxidative stress because their membranes contain high concentrations of polyunsaturated fatty acids, which are highly susceptible to peroxidation (17). Copper and Zn form the Cu-Zn superoxide SOD, which is responsible for the dismutation of superoxide radicals to hydrogen peroxide in the cytosol (54). Mn improves the immune response due to its essential role in the elimination of superoxide radicals produced during inflammation (55). In addition to an antioxidant role, Zn facilitate proper innate and adaptive immune functions by promoting cell replication and proliferation (17). Similarly, Batistel et al. (7) reported that supplementation of periparturient cows with complex trace elements (Zn, Mn, Cu and Co) from day -30 to +30 improved neutrophil activity and early uterine tissue recovery by reducing inflammation and enhancing antioxidant response. In accordance with our findings, several studies have reported that administration of Se and vitamin E promotes growth, improves immune response, and reduces the incidence of infectious diseases in calves by reducing perinatal oxidative stress (56, 57). Vitamin E improves T-cell mediated function by promoting membrane integrity, positively modulating signalling events in T-cells and reducing the production of T-cell suppressing factors such as PGE2 (58). In addition, vitamin E improves neutrophil phagocytic activity and humoral immunity in transition cows and their calves by acting as a chain-breaking antioxidant and protecting the cell membrane from lipid peroxidation (59). The major antioxidant functions of selenium are mediated by selenoproteins such as glutathione peroxidase and thioredoxin reductase. Selenoproteins play a critical role in maintaining optimal immune function by preventing and mitigating oxidative stress, and optimizing the inflammatory response (57). Volpato et al. (56) reported that injectable administration of Se together with vitamins (A and E) improves immune response and minimizes health disorders in newborn heifers. Therefore, the higher phagocytosis capacity of neutrophils and lymphocyte proliferation of the treated cows and their calves could be attributed to the improved antioxidant status of these animals.

TLRs play an important role in immune response orchestration and have multiple and diverse effects on neutrophils, including cytokine production, ROS generation, priming, receptor expression, chemotaxis, and phagocytosis (60). In the present study, the mRNA expression of TLR2 and TLR4 on blood neutrophils was higher in the control group at a specific week’s interval during the transition period, which is in accordance with the findings of Crookenden et al. (9). Higher TLR expression in control groups indicates higher stress levels and stronger inflammatory response as compared to the treated groups. Similarly, Sadeghi et al. (61) reported that vitamin D supplementation lowers the expression of TLR2, TLR4, and TLR9 in monocytes during autoimmune disease. El-Zayat et al. (62) reported that TLR4 activation during stressful physiological conditions causes an increase in NF-kB pro-inflammatory response. However, if these changes are not controlled and allowed to persist, it can cause a slew of health issues for the animal. In line with our findings, previous studies reported lower expression of GR-α on neutrophils at calving and at the time of disease diagnosis, which was associated with a decrease in the phagocytic activity, chemotaxis, and relative mRNA expression of some important neutrophil receptors, along with a concomitant rise in plasma cortisol (8, 41). CD62L and CD11b are cell adhesion molecules that contribute to adhesion, migration, and signal transduction along the microvascular endothelial walls (15). An effective response to an inflammatory process necessitates the opposite modulation of these two molecules, which results in the upregulation of CD11b on blood neutrophils and the rapid shedding of CD62L (41), which was more evident in micronutrient injected groups indicating, the role of micronutrients in preventing the impairment immune cell functions. Lower expression of inflammatory mediators such as CXCRs and TLRs on neutrophils may be due to altered micronutrient availability (63), implying that injecting micronutrients during this stressful period may control inflammatory response and limit tissue damage and oxidative stress in transition cows and their newborn calves. Besides, the severity and persistence of reduced neutrophil GR-α expression in control cows indicated that homeostasis was compromised, and oxidative stress persisted for longer days as compared to the treated group. Newborn calves are also exposed to tremendous oxidative stress in the first days of life, which is associated with an increased inflammatory response and poor immune function, especially if the calves do not receive high-quality colostrum (13). The lower expression of genes associated with inflammation in calves in the micronutrient-injected group, particularly in the MMMV groups, therefore highlights the importance of antioxidants in reducing oxidative stress and inflammatory response in newborn calves. Similarly, Teixeira et al. (64) reported that injection of a multimineral preparation containing Zn, Mn, Se and Co in the early postnatal period improved neutrophil function and antioxidant capacity in calves, while reducing oxidative stress and disease incidence.

The inflammatory response bridges the gap between innate and adaptive immunity, and is influenced by the availability of vitamins A, C, E, and B6, as well as iron, zinc, and copper (17, 20). Previous studies have shown that pro and anti-inflammatory cytokines are released simultaneously, and timely release of the anti-inflammatory cytokine is necessary for the control of the pro-inflammatory cytokines and results in the resolution of inflammatory conditions. Anti-inflammatory cytokines such as IL-4 and IL-10 inhibit the production of pro-inflammatory molecules like, TNF-α, IL-1β, IL-12, and IFN-γ and limit tissue damage and oxidative stress (65). The significant increase in pro-inflammatory cytokines in control cows may be attributed to a higher degree of calving stress and inflammatory condition as compared to the micronutrient injected cows which are in accordance with previous reports showing higher levels of pro-inflammatory cytokines in cows suffering from postpartum reproductive problems (4, 5). Lower levels of IL-4 and IL-10 in control cows may be due to an increased state of inflammatory immune response compared to micronutrient treated groups. Similarly, Grewal et al. (66) demonstrated that the expression of IL-10 in PBMC of periparturient dairy cows was highest on day 21 prepartum and reached the lowest levels on the day of calving and attributed this to the increased production of pro-inflammatory cytokines including IL-1β, IL-6 and TNF-α. Bochniarz et al. (67) also reported lower levels of IL-10 in the serum and milk of cows with subclinical mastitis cows. Newborn calves are subjected to a remarkable stress and inflammatory response during the first three weeks of the neonatal period, as they are immunologically naive at birth and require the transition from passive to active immunity due to exposure to environmental microorganisms (68). Colostrum is rich in immunoglobulins and many bioactive compounds that are essential for countering postnatal challenges by enhancing the immune response and stimulating intestinal maturation in newborn calves (12, 69). In the present study, the improved immune quality of colostrum and the higher concentration of plasma TIG in calves injected with micronutrients could explain the lower inflammatory response and enhanced immune homeostasis in these groups compared to the control. In agreement with our results, Jacometo et al. (70) reported that the administration of organic trace elements (Zn, Mn, Cu and Co) to periparturient dairy cows promotes the innate immune response of their newborn calves by reducing the expression of inflammatory mediators in blood neutrophils. Moreover, feeding vitamin D3 decreased the concentrations of inflammatory cytokines (IL-1β, IL-6 and TNF-α), stress indicators (cortisol and haptoglobin) and oxidative stress in weaning calves (71).

Maternal mineral and vitamin status influences not only the health of the dam, but also that of the offspring (11, 72). Optimum availability of these essential nutrients influences fetal development *via* placental transfer of nutrients to the fetus and later affects calf health *via* colostrum and milk quality (11). Colostrum quality is determined by the concentration of colostrum IgG, which should be higher than 50 mg/ml for good quality colostrum (69, 73). In the present study, the concentration of TIG in colostrum was significantly higher in the MMMV group at day 0 (104.41 mg/ml) compared to other groups. Zinc is essential for an optimal humoral immune response, the uptake of Ig by the epithelial cells of the mammary gland, the secretion of Ig in the colostrum and the uptake by the calf (10). Roshanzamir et al. (14) supplemented Cu, Mn, and Zn for two months before calving and reported increased antioxidant capacity and immunoglobulins in calf serum. Pekmezci and Cakiroglu (74) demonstrated that supplementation of vitamin E during the first two weeks after birth increased the concentrations of IgM and IgG in Jersey calves and attributed this to the ability of vitamin E to stimulate synthesis and secretion of immunoglobulins by B lymphocytes. The newborn calf requires a large amount of colostrum IgG in the first hours of life to ensure effective transfer of immunity (69). Successful passive immunity transfer is defined as a serum IgG concentration greater than 10 mg/ml in the first 24 hours after birth (75). Recently, this definition has been revised as follows: excellent, greater than 25 mg/ml; good 18-24.9 mg/ml; moderate, 10-17.9 mg/ml; and poor, less than 10 mg/ml (76). Although the maternal colostral antibodies could reduce the efficacy of the vaccine in the newborn calves, it protects them from infectious diseases that could affect their performance and lead to high mortality (77, 78). Palomares et al. (79) indicated that injectable trace elements may be a promising means of improving antibody titer, immune cell proliferation and immune memory to modified live viral vaccines in dairy calves. In our study, TIG levels in the MM and MMMV group indicate excellent passive immunity transmission and are within the recommended optimal range for vaccination programs (76, 77).

In the present study, of the three treatments used, MMMV showed the best effect on periparturient cows and their calves. The MMMV group showed the lowest inflammation and oxidative stress, as well as the highest antioxidant capacity and immune response compared to the other groups. In addition, the colostrum of the MMMV group had excellent quality in terms of TIG content, and the calves of the MMMV group had the best rate of passive transfer of immunity.

## 5 Conclusions

According to our results, the combined injection of minerals and vitamins had an optimal effect on the health status of the animals compared to the injection of either minerals or vitamins. Therefore, an adequate supply of vitamins and minerals is essential for maintaining a balanced immune response by regulating the inflammatory response and reducing oxidative stress in periparturient cows and their offspring. In addition, the increased TIG concentration and improved innate and adaptive immune response in calves of treated cows, especially in the MMMV group, reflect improved colostrum and milk quality, which improves calf health and survival. Further studies are needed to determine the optimal dosage and best combination of trace elements and vitamins based on the animal’s health status, productivity, and physiological stage to ensure optimal health and productivity without side effects.

## Data availability statement

The datasets presented in this study can be found in online repositories. The names of the repository/repositories and accession number(s) can be found in the article/**Supplementary Material**.

## Ethics statement

The animal study was reviewed and approved vide order no. 42-IAEC-18-6 from Institute Animal Ethics Committee (IAEC) according to the Committee for the Purpose of Control and Supervision of Experiments on Animals (CPCSEA), laid down by the Government of India.

## Author contributions

YS, MA, and AD conceived and designed the study. YS performed all the experiments. YS, MA, and AD analyzed the data and interpreted the results. YS and MA prepared the figures, tables, wrote and revised the manuscript. AD supervised the project and revised the manuscript. All authors contributed to the article and approved the submitted version.
