# Supplementary material for: Repeated injection of multivitamins and multiminerals during the transition period enhances immune response by suppressing inflammation and oxidative stress in cows and their calves
Source: Front Immunol. 2023 Feb 9;14:1059956. doi: 10.3389/fimmu.2023.1059956 (PMC9950815; doi:10.3389/fimmu.2023.1059956)
Supplement: Supplementary file 1 [file Table_1.docx]

Supplementary Material

# Supplementary Tables

**Supplementary Table 1.** Viability of neutrophils and lymphocytes (percentage) of different groups of periparturient Karan Fries cows, i.e. Control (Basal Diet, BD), MM (BD + Multi-mineral), MV (BD + Multi-vitamin), and MMMV (BD + Multimineral and Multivitamins).

| **Items/ Days** | **Groups** | | | |
| --- | --- | --- | --- | --- |
|  | **Control** | **MM** | **MV** | **MMMV** |
| **Viability of neutrophils** | | | | |
| **-30** | 97.66±1.33 | 97.66±1.85 | 98.00±1.00 | 98.00±1.00 |
| **-15** | 96.33±1.76 | 97.33±0.66 | 97.00±1.15 | 97.00±1.52 |
| **-7** | 96.00±1.52 | 96.66±1.85 | 96.33±1.45 | 96.33±1.66 |
| **0** | 95.33±1.76 | 96.00±1.15 | 95.66±1.45 | 96.00±1.52 |
| **7** | 94.66±2.40 | 95.66±1.20 | 96.00±1.73 | 96.66±1.85 |
| **15** | 97.66±0.88 | 97.00±1.52 | 96.33±1.76 | 97.00±1.52 |
| **30** | 98.00±1.00 | 98.00±1.00 | 97.33±1.66 | 98.33±0.66 |
| **Overall Mean** | **96.52±1.51** | **96.90±1.25** | **96.66±1.32** | **97.04±1.29** |
| **Viability of Lymphocytes** | | | | |
| **-30** | 98.33±0.66 | 98.00±0.57 | 97.00±1.73 | 98.00±1.00 |
| **-15** | 97.66±1.33 | 97.33±1.20 | 96.33±1.45 | 97.00±1.15 |
| **-7** | 96.33±1.20 | 96.66±1.45 | 96.66±1.85 | 96.66±1.45 |
| **0** | 96.00±1.52 | 96.00±1.52 | 96.33±1.20 | 96.00±2.08 |
| **7** | 96.66±0.88 | 96.33±1.45 | 97.00±0.57 | 97.00±1.15 |
| **15** | 97.33±1.20 | 97.66±0.88 | 97.66±0.88 | 97.66±0.88 |
| **30** | 97.33±1.66 | 98.00±1.00 | 97.66±0.88 | 98.00±1.52 |
| **Overall Mean** | **97.09±1.13** | **97.14±1.09** | **96.95±1.13** | **97.19±1.21** |

**Supplementary Table 2.** Viability of neutrophils and lymphocytes (percentage) in calves born to different groups of periparturient Karan Fries cows, i.e. Control (Basal Diet, BD), MM (BD + Multi-mineral), MV (BD + Multi-vitamin), and MMMV (BD + Multimineral and Multivitamins)

| **Items/ Days** | **Groups** | | | |
| --- | --- | --- | --- | --- |
|  | **Control** | **MM** | **MV** | **MMMV** |
| **Viability of neutrophils** | | | | |
| **0** | 95.66±0.88 | 96.00±1.73 | 95.33±1.33 | 96.33±1.45 |
| **3** | 96.00±1.52 | 97.33±0.88 | 96.66±0.66 | 97.00±1.73 |
| **7** | 96.33±1.66 | 97.00±1.15 | 97.00±1.52 | 97.66±1.33 |
| **15** | 97.00±1.52 | 97.66±1.20 | 97.66±0.66 | 97.33±1.20 |
| **30** | 97.00±1.73 | 98.00±1.15 | 97.33±1.20 | 98.00±1.00 |
| **45** | 98.33±0.66 | 98.00±0.57 | 98.33±0.88 | 98.66±0.88 |
| **Overall Mean** | **96.72±1.28** | **97.33±1.06** | **97.05±1.07** | **97.5±1.17** |
| **Viability of Lymphocytes** | | | | |
| **0** | 95.66±0.88 | 96.66±1.85 | 96.33±1.45 | 96.66±0.88 |
| **3** | 96.00±1.15 | 97.00±1.00 | 97.66±0.88 | 97.00±1.52 |
| **7** | 97.00±1.52 | 97.23±0.90 | 98.00±1.15 | 97.33±0.88 |
| **15** | 96.33±1.45 | 98.00±1.15 | 97.66±0.88 | 98.00±0.57 |
| **30** | 97.66±1.20 | 97.33±0.88 | 97.33±0.88 | 98.33±1.20 |
| **45** | 98.66±0.88 | 97.33±0.88 | 98.33±0.66 | 98.66±0.88 |
| **Overall Mean** | **96.83±1.20** | **97.26±1.00** | **97.55±0.93** | **97.66±0.97** |

**Supplementary Table 3.** Blood differential leucocytes count of different groups of periparturient Karan Fries cows, i.e. Control (Basal Diet, BD), MM (BD + Multi-mineral), MV (BD + Multi-vitamin), and MMMV (BD + Multimineral and Multivitamins)

| **Items/ Days** | **Groups** | | | | **RMSE** | **P-value** | | |
| --- | --- | --- | --- | --- | --- | --- | --- | --- |
|  | **Control** | **MM** | **MV** | **MMMV** |  | **D** | **Gr** | **D×Gr** |
| **Band neutrophils percentage** | | | | | | | | |
| **-30** | 1.33±0.33^ab^ | 0.66±0.33^a^ | 1.00±0.57^a^ | 0.66±0.33^a^ | 0.988 | ≤0.001 | ≤0.001 | ns |
| **-15** | 2.00±0.57^ab^ | 1.00±0.57^a^ | 2.00±0.57^ab^ | 0.66±0.66^a^ |  |  |  |  |
| **-7** | 4.00±0.57^bcB^ | 2.33±0.33^abA^ | 3.00±0.57^abAB^ | 1.66±0.33^aA^ |  |  |  |  |
| **0** | 7.66±0.88^d^ | 6.00±0.57^d^ | 6.00±1.15^c^ | 5.33±0.88^c^ |  |  |  |  |
| **7** | 5.33±0.66^cd^ | 5.00±0.57^cd^ | 4.66±0.66^bc^ | 4.00±0.57^bc^ |  |  |  |  |
| **15** | 3.33±0.88^abc^ | 3.66±0.33^bc^ | 2.66±0.33^ab^ | 2.33±0.33^ab^ |  |  |  |  |
| **30** | 1.00±0.57^a^ | 0.66±0.33^a^ | 0.66±0.33^a^ | 1.00±0.57^a^ |  |  |  |  |
| **Overall Mean** | **3.52±0.53^B^** | **2.76±0.47^AB^** | **2.85±0.44^AB^** | **2.23±0.41^A^** |  |  |  |  |
| **Segmented neutrophils percentage** | | | | | | | | |
| **-30** | 98.86±0.35^c^ | 99.34±0.32^d^ | 99.01±0.46^c^ | 99.39±0.32^c^ | 0.920 | ≤0.001 | ≤0.001 | ns |
| **-15** | 98.33±0.44^c^ | 98.83±0.52^d^ | 98.30±0.41^c^ | 99.36±0.49^c^ |  |  |  |  |
| **-7** | 96.03±0.60^bA^ | 97.55±0.49^cdAB^ | 97.31±0.59^bcAB^ | 98.04±0.30^cB^ |  |  |  |  |
| **0** | 92.40±0.90^a^ | 93.89±0.43^a^ | 94.03±1.12^a^ | 94.53±0.81^a^ |  |  |  |  |
| **7** | 94.70±0.47^b^ | 95.07±0.58^ab^ | 95.53±0.51^ab^ | 96.03±0.26^ab^ |  |  |  |  |
| **15** | 96.76±0.88^bc^ | 96.63±0.42^bc^ | 97.39±0.26^bc^ | 97.75±0.14^bc^ |  |  |  |  |
| **30** | 99.10±0.20^c^ | 99.41±0.30^d^ | 99.43±0.29^c^ | 98.95±0.53^c^ |  |  |  |  |
| **Overall Mean** | **96.63±0.55^A^** | **97.30±0.46^AB^** | **97.29±0.44^AB^** | **97.76±0.41^B^** |  |  |  |  |
| **Total neutrophils percentage** | | | | | | | | |
| **-30** | 30.66±2.02^a^ | 31.00±1.15^a^ | 31.00±1.15^a^ | 29.66±1.45^a^ | 2.718 | ≤0.001 | ≤0.001 | ns |
| **-15** | 34.66±1.76^ab^ | 31.00±3.05^a^ | 30.66±1.45^a^ | 31.33±0.88^a^ |  |  |  |  |
| **-7** | 36.66±1.85^abB^ | 33.66±1.20^aAB^ | 34.00±1.73^abAB^ | 30.33±1.76^aA^ |  |  |  |  |
| **0** | 41.33±1.76^bB^ | 36.00±1.00^aA^ | 38.00±1.73^bAB^ | 34.00±0.57^aA^ |  |  |  |  |
| **7** | 38.33±1.20^abB^ | 33.33±1.76^aAB^ | 34.66±0.88^abAB^ | 31.00±0.57^aA^ |  |  |  |  |
| **15** | 35.33±1.76^ab^ | 32.33±2.02^a^ | 33.00±1.52^ab^ | 30.33±1.45^a^ |  |  |  |  |
| **30** | 33.00±1.52^a^ | 29.00±1.73^a^ | 31.00±1.15^a^ | 28.00±1.52^a^ |  |  |  |  |
| **Overall Mean** | **35.71±0.90^C^** | **32.33±0.74^AB^** | **33.19±0.70^B^** | **30.66±0.54^A^** |  |  |  |  |
| **Lymphocytes percentage** | | | | | | | | |
| **-30** | 66.33±1.20^c^ | 66.00±1.15^b^ | 67.66±0.88^b^ | 67.33±0.66^b^ | 2.380 | ≤0.001 | ≤0.001 | ns |
| **-15** | 64.00±1.73^bc^ | 65.66±1.20^b^ | 66.66±1.33^b^ | 67.33±0.88^b^ |  |  |  |  |
| **-7** | 61.33±1.45^abc^ | 63.00±1.52^ab^ | 63.66±1.45^ab^ | 65.00±1.15^ab^ |  |  |  |  |
| **0** | 56.00±1.15^aA^ | 58.66±1.20^aAB^ | 59.66±1.20^aAB^ | 61.00±1.52^aB^ |  |  |  |  |
| **7** | 58.66±2.02^ab^ | 62.00±1.73^ab^ | 63.00±1.52^ab^ | 63.00±1.15^ab^ |  |  |  |  |
| **15** | 61.33±1.20^abc^ | 64.00±1.52^ab^ | 64.66±1.20^ab^ | 65.66±1.76^ab^ |  |  |  |  |
| **30** | 63.66±1.76^bc^ | 66.66±1.66^b^ | 67.66±1.45^b^ | 67.00±0.57^b^ |  |  |  |  |
| **Overall Mean** | **61.61±0.86^A^** | **63.71±0.73^B^** | **64.71±0.73^B^** | **65.19±0.62^B^** |  |  |  |  |
| **Monocytes percentage** | | | | | | | | |
| **-30** | 3.00±0.57^a^ | 3.66±0.88^a^ | 1.66±0.33^a^ | 3.00±0.57^ab^ | 1.052 | 0.0003 | ≤0.001 | ns |
| **-15** | 1.66±0.33^a^ | 3.33±0.66^a^ | 2.66±0.66^a^ | 2.00±0.57^a^ |  |  |  |  |
| **-7** | 4.00±0.57^aAB^ | 3.33±0.33^aAB^ | 2.33±0.33^aA^ | 4.66±0.66^abcB^ |  |  |  |  |
| **0** | 4.33±0.88^aAB^ | 5.33±0.33^aB^ | 2.33±0.33^aA^ | 5.66±0.88^bcB^ |  |  |  |  |
| **7** | 3.00±0.57^aA^ | 4.66±0.66^aAB^ | 2.33±0.88^aA^ | 6.00±0.57^cB^ |  |  |  |  |
| **15** | 3.33±0.33^a^ | 3.66±0.33^a^ | 2.66±0.88^a^ | 4.33±0.33^abc^ |  |  |  |  |
| **30** | 3.33±0.66^aAB^ | 4.66±0.66^aAB^ | 2.00±0.57^aA^ | 5.33±0.66^bcB^ |  |  |  |  |
| **Overall Mean** | **3.23±0.25^B^** | **4.09±0.24^C^** | **2.28±0.20^A^** | **4.42±0.36^C^** |  |  |  |  |

Means with different small superscripts (a, b, c and d) and capital superscripts (A, B and C) differ significantly (P<0.05) in a column and in a row, respectively. **RMSE:** Root Mean Square Error, **D**: Days of sampling, **Gr**: Treatment groups.

**Supplementary Table 4.** Blood differential leucocytes count in calves born to different groups of periparturient Karan Fries cows, i.e. Control (Basal Diet, BD), MM (BD + Multi-mineral), MV (BD + Multi-vitamin), and MMMV (BD + Multimineral and Multivitamins).

| **Items/ Days** | **Groups** | | | | **RMSE** | **P-value** | | |
| --- | --- | --- | --- | --- | --- | --- | --- | --- |
|  | **Control** | **MM** | **MV** | **MMMV** |  | **D** | **Gr** | **D×Gr** |
| **Band neutrophils percentage** | | | | | | | | |
| **0** | 11.00±0.57^bC^ | 7.33±0.66^cAB^ | 5.66±1.20^bA^ | 10.00±1.15^bBC^ | 1.258 | ≤0.001 | ≤0.001 | ≤0.001 |
| **3** | 8.00±1.00^bcB^ | 4.66±0.88^bcAB^ | 3.66±0.66^abA^ | 2.33±0.88^aA^ |  |  |  |  |
| **7** | 10.33±0.88^cC^ | 5.66±0.33^cB^ | 3.00±0.57^abA^ | 2.66±0.66^aA^ |  |  |  |  |
| **15** | 4.00±0.57^aC^ | 2.00±0.57^abB^ | 3.33±0.88^abBC^ | 0.66±0.33^aA^ |  |  |  |  |
| **30** | 3.66±0.33^aB^ | 1.33±0.66^aA^ | 2.33±0.33^abAB^ | 1.33±0.66^aA^ |  |  |  |  |
| **45** | 3.00±0.57^a^ | 1.66±0.88^a^ | 1.66±0.66^a^ | 1.66±0.88^a^ |  |  |  |  |
| **Overall Mean** | **6.00±0.68^B^** | **3.77±0.59^A^** | **3.27±0.40^A^** | **2.50±0.51^A^** |  |  |  |  |
| **Segmented neutrophils percentage** | | | | | | | | |
| **0** | 89.00±0.57^aA^ | 92.66±0.66^aAB^ | 94.33±1.20^aB^ | 90.00±1.15^aA^ | 1.476 | ≤0.001 | ≤0.001 | ≤0.001 |
| **3** | 92.00±1.00^abA^ | 95.33±0.88^abcAB^ | 96.33±0.66^aB^ | 97.66±0.88^bB^ |  |  |  |  |
| **7** | 89.66±0.88^aA^ | 94.33±0.33^abB^ | 97.00±0.57^aC^ | 97.33±0.66^bC^ |  |  |  |  |
| **15** | 96.00±0.57^cA^ | 98.00±0.57^cdAB^ | 96.66±0.88^aAB^ | 99.33±0.33^bB^ |  |  |  |  |
| **30** | 96.33±0.33^c^ | 98.66±0.66^d^ | 97.66±0.33^a^ | 98.66±0.66^b^ |  |  |  |  |
| **45** | 95.00±1.15^bc^ | 96.33±0.88^bcd^ | 96.66±1.45^a^ | 96.00±1.52^b^ |  |  |  |  |
| **Overall Mean** | **93.00±0.76^A^** | **95.88±0.55^B^** | **96.44±0.40^B^** | **96.50±0.81^B^** |  |  |  |  |
| **Total neutrophils percentage** | | | | | | | | |
| **0** | 48.00±1.15^dC^ | 43.60±0.88^dB^ | 42.60±1.45^cAB^ | 39.00±0.57^cA^ | 2.131 | ≤0.001 | ≤0.001 | ns |
| **3** | 42.66±1.45^cdC^ | 38.3±0.66^cAB^ | 40.30±0.66^bcBC^ | 35.30±0.66^bcA^ |  |  |  |  |
| **7** | 38.66±1.76^bc^ | 36.00±0.57^bc^ | 35.30±1.45^ab^ | 34.60±0.88^bc^ |  |  |  |  |
| **15** | 36.00±1.15^ab^ | 35.00±1.15^bc^ | 34.00±2.08^a^ | 31.60±0.88^ab^ |  |  |  |  |
| **30** | 31.00±1.15^a^ | 30.60±0.88^a^ | 31.60±1.20^a^ | 28.60±0.88^a^ |  |  |  |  |
| **45** | 34.33±1.76^ab^ | 32.00±1.52^ab^ | 31.00±0.57^a^ | 31.3±2.02^ab^ |  |  |  |  |
| **Overall Mean** | **38.44±1.44^C^** | **35.94±1.09^B^** | **35.83±1.14^B^** | **33.44±0.89^A^** |  |  |  |  |
| **Lymphocytes percentage** | | | | | | | | |
| **0** | 50.66±0.88^a^ | 52.66±1.20^a^ | 55.00±1.73^a^ | 55.00±1.15^a^ | 2.220 | ≤0.001 | ≤0.001 | ns |
| **3** | 54.66±0.66^aA^ | 54.66±0.88^abA^ | 59.00±0.57^abB^ | 58.33±1.76^abAB^ |  |  |  |  |
| **7** | 59.33±1.20^b^ | 59.33±1.76^bc^ | 60.00±1.52^ab^ | 60.00±1.52^abc^ |  |  |  |  |
| **15** | 60.33±1.66^b^ | 62.66±2.18^c^ | 63.00±1.15^b^ | 64.33±1.45^bc^ |  |  |  |  |
| **30** | 61.66±0.88^b^ | 62.00±0.57^c^ | 63.00±1.00^b^ | 64.00±1.15^bc^ |  |  |  |  |
| **45** | 62.00±0.57^b^ | 63.00±1.15^c^ | 63.66±1.45^b^ | 65.33±0.88^c^ |  |  |  |  |
| **Overall Mean** | **58.11±1.05^A^** | **59.05±1.08^AB^** | **60.61±0.85^AB^** | **61.16±1.01^B^** |  |  |  |  |
| **Monocytes percentage** | | | | | | | | |
| **0** | 2.00±0.57^aA^ | 3.66±0.66^aAB^ | 3.00±0.57^abAB^ | 5.00±0.57^abcB^ | 1.142 | ≤0.001 | 0.0002 | 0.00015 |
| **3** | 3.00±1.15^aAB^ | 7.00±0.57^bcB^ | 2.00±0.57^aA^ | 4.66±0.88^abcAB^ |  |  |  |  |
| **7** | 2.66±0.33^aA^ | 4.66±0.33^abB^ | 5.33±0.88^bB^ | 7.33±0.66^cC^ |  |  |  |  |
| **15** | 4.00±0.57^a^ | 3.00±0.57^a^ | 3.00±0.57^ab^ | 4.00±0.57^ab^ |  |  |  |  |
| **30** | 7.00±0.57^b^ | 7.66±0.33^c^ | 5.66±0.66^b^ | 7.00±1.00^bc^ |  |  |  |  |
| **45** | 4.00±0.57^a^ | 5.00±1.00^ab^ | 5.66±0.33^b^ | 3.66±0.33^a^ |  |  |  |  |
| **Overall Mean** | **3.77±0.45^A^** | **5.16±0.45^B^** | **4.11±0.41^A^** | **5.27±0.41^B^** |  |  |  |  |

Means with different small superscripts (a, b, c and d) and capital superscripts (A, B and C) differ significantly (P<0.05) in a column and in a row, respectively. **RMSE:** Root Mean Square Error, **D**: Days of sampling, **Gr**: Treatment groups.

**Supplementary Table 5.** Neutrophil phagocytic activity (PA) and lymphocytes proliferation level of different groups of periparturient Karan Fries cows, i.e. Control (Basal Diet, BD), MM (BD + Multi-mineral), MV (BD + Multi-vitamin), and MMMV (BD + Multimineral and Multivitamins).

| **Items/ Days** | **Groups** | | | | **RMSE** | **P-value** | | |
| --- | --- | --- | --- | --- | --- | --- | --- | --- |
|  | **Control** | **MM** | **MV** | **MMMV** |  | **D** | **Gr** | **D×Gr** |
| **Phagocytic activity (PA) of neutrophils** | | | | | | | | |
| **-30** | 1.37±0.06^e^ | 1.41±0.10^c^ | 1.40±0.21^de^ | 1.45±0.08^cd^ | 0.097 | ≤0.001 | ≤0.001 | ns |
| **-15** | 1.15±0.04^cdA^ | 1.31±0.03^cAB^ | 1.39±0.06^deB^ | 1.42±0.12^cdB^ |  |  |  |  |
| **-7** | 0.71±0.05^bA^ | 0.93±0.07^abB^ | 1.05±0.02^bcB^ | 1.15±0.04^abB^ |  |  |  |  |
| **0** | 0.48±0.03^aA^ | 0.73±0.02^aB^ | 0.70±0.08^aB^ | 0.93±0.06^aB^ |  |  |  |  |
| **7** | 0.69±0.04^bA^ | 0.95±0.07^abB^ | 0.91±0.04^bB^ | 1.21±0.05^bcC^ |  |  |  |  |
| **15** | 0.99±0.03^cA^ | 1.20±0.11^bcAB^ | 1.20±0.05^cdAB^ | 1.23±0.07^bcB^ |  |  |  |  |
| **30** | 1.26±0.10^deA^ | 1.35±0.09^cAB^ | 1.43±0.00^eAB^ | 1.57±0.06^dB^ |  |  |  |  |
| **Overall Mean** | **0.95±0.07^A^** | **1.12±0.05^B^** | **1.15±0.06^B^** | **1.28±0.05^C^** |  |  |  |  |
| **Lymphocytes proliferation response** | | | | | | | | |
| **-30** | 1.49±0.07^c^ | 1.51±0.07^c^ | 1.52±0.10^c^ | 1.59±0.13^b^ | 0.1746 | ≤0.001 | ≤0.001 | ns |
| **-15** | 0.95±0.13^ab^ | 1.06±0.13^ab^ | 1.02±0.14^ab^ | 1.20±0.13^ab^ |  |  |  |  |
| **-7** | 0.73±0.08^abA^ | 0.99±0.06^abAB^ | 0.89±0.12^abAB^ | 1.27±0.08^abB^ |  |  |  |  |
| **0** | 0.51±0.11^aA^ | 0.70±0.05^aAB^ | 0.70±0.05^aAB^ | 1.03±0.10^aB^ |  |  |  |  |
| **7** | 0.62±0.10^abA^ | 0.72±0.08^aAB^ | 0.60±0.08^aA^ | 1.07±0.08^aB^ |  |  |  |  |
| **15** | 0.79±0.05^abA^ | 1.05±0.07^abAB^ | 0.97±0.12^abAB^ | 1.37±0.11^abB^ |  |  |  |  |
| **30** | 1.04±0.08^b^ | 1.29±0.07^bc^ | 1.25±0.10^bc^ | 1.41±0.09^ab^ |  |  |  |  |
| **Overall Mean** | **0.87±0.07^A^** | **1.05±0.06^B^** | **0.99±0.07^AB^** | **1.28±0.05^C^** |  |  |  |  |

Means with different small superscripts (a, b, c and d) and capital superscripts (A, B and C) differ significantly (P<0.05) in a column and in a row, respectively. **RMSE:** Root Mean Square Error, **D**: Days of sampling, **Gr**: Treatment groups.

**Supplementary Table 6.** Neutrophil phagocytic activity (PA) and lymphocytes proliferation level in calves born to different groups of periparturient Karan Fries cows, i.e. Control (Basal Diet, BD), MM (BD + Multi-mineral), MV (BD + Multi-vitamin), and MMMV (BD + Multimineral and Multivitamins)

| **Items/ Days** | **Groups** | | | | **RMSE** | **P-value** | | |
| --- | --- | --- | --- | --- | --- | --- | --- | --- |
|  | **Control** | **MM** | **MV** | **MMMV** |  | **D** | **Gr** | **D×Gr** |
| **Neutrophil phagocytic activity (PA)** | | | | | | | | |
| **0** | 0.18±0.02^aA^ | 0.29±0.03^aAB^ | 0.21±0.03^aA^ | 0.423±0.04^aB^ | 0.164 | ≤0.001 | ≤0.001 | ns |
| **3** | 0.23±0.03^aA^ | 0.41±0.03^aBC^ | 0.30±0.06^aAB^ | 0.52±0.03^abC^ |  |  |  |  |
| **7** | 0.44±0.08^abA^ | 0.73±0.05^abAB^ | 0.54±0.07^bAB^ | 0.78±0.07^bB^ |  |  |  |  |
| **15** | 0.74±0.07^bcA^ | 1.21±0.18^bcAB^ | 0.86±0.05^cAB^ | 1.34±0.11^cB^ |  |  |  |  |
| **30** | 1.04±0.13^c^ | 1.37±0.17^c^ | 1.30±0.04^d^ | 1.51±0.08^c^ |  |  |  |  |
| **45** | 1.13±0.14^c^ | 1.33±0.20^c^ | 1.29±0.05^b^ | 1.50±0.06^c^ |  |  |  |  |
| **Overall Mean** | **0.63±0.09^A^** | **0.89±0.11^B^** | **0.75±0.10^A^** | **1.01±0.11^B^** |  |  |  |  |
| **Lymphocytes proliferation response** | | | | | | | | |
| **0** | 0.52±0.04^a^ | 0.52±0.05^a^ | 0.55±0.04^a^ | 0.58±0.03^a^ | 0.121 | ≤0.001 | ≤0.001 | ns |
| **3** | 0.59±0.07^abA^ | 0.82±0.04^bB^ | 0.62±0.03^aA^ | 1.05±0.04^bC^ |  |  |  |  |
| **7** | 0.87±0.03^bc^ | 1.00±0.03^bc^ | 0.94±0.06^b^ | 1.10±0.10^b^ |  |  |  |  |
| **15** | 0.99±0.08^c^ | 1.26±0.10^cd^ | 1.18±0.09^c^ | 1.40±0.12^c^ |  |  |  |  |
| **30** | 1.13±0.09^cA^ | 1.36±0.07^dAB^ | 1.44±0.06^dAB^ | 1.59±0.06^cB^ |  |  |  |  |
| **45** | 1.16±0.08^cA^ | 1.42±0.07^dB^ | 1.47±0.03^dB^ | 1.60±0.03^cB^ |  |  |  |  |
| **Overall Mean** | **0.88±0.06^A^** | **1.06±0.08^B^** | **1.03±0.09^B^** | **1.22±0.09^B^** |  |  |  |  |

Means with different small superscripts (a, b, c and d) and capital superscripts (A, B and C) differ significantly (P<0.05) in a column and in a row, respectively. **RMSE:** Root Mean Square Error, **D**: Days of sampling, **Gr**: Treatment groups.

**Supplementary Table 7.** Relative mRNA expression of toll-like, chemokine and glucocorticoid receptors in blood neutrophils of different groups of periparturient Karan Fries cows, i.e. Control (Basal Diet, BD), MM (BD + Multi-mineral), MV (BD + Multi-vitamin), and MMMV (BD + Multimineral and Multivitamins).

| **Items/ Days** | **Groups** | | | | **RMSE** | **P-value** | | |
| --- | --- | --- | --- | --- | --- | --- | --- | --- |
|  | **Control** | **MM** | **MV** | **MMMV** |  | **D** | **Gr** | **D×Gr** |
| **Toll-like receptor 2 (TLR2)** | | | | | | | | |
| **-30** | 1.00±0.00^a^ | 1.00±0.00^a^ | 1.00±0.00^a^ | 1.00±0.00^a^ | 0.122 | ≤0.001 | ≤0.001 | 0.0029 |
| **-15** | 1.79±0.05^bB^ | 1.30±0.12^bA^ | 1.32±0.04^bA^ | 1.13±0.02^abA^ |  |  |  |  |
| **-7** | 1.64±0.05^bB^ | 1.45±0.06^bcAB^ | 1.48±0.05^bcAB^ | 1.27±0.09^abA^ |  |  |  |  |
| **0** | 2.29±0.07^dC^ | 1.92±0.03^dAB^ | 2.05±0.04^dB^ | 1.75±0.03^cA^ |  |  |  |  |
| **7** | 2.16±0.13^cdB^ | 1.62±0.05^cA^ | 1.69±0.04^bcA^ | 1.41±0.07^bA^ |  |  |  |  |
| **15** | 1.89±0.04^bcB^ | 1.36±0.08^bcA^ | 1.6±0.15^bcAB^ | 1.25±0.04^abA^ |  |  |  |  |
| **30** | 1.55±0.09^bB^ | 1.23±0.06^abAB^ | 1.35±0.05^bAB^ | 1.19±0.08^abA^ |  |  |  |  |
| **Overall Mean** | **1.76±0.09^C^** | **1.41±0.06^B^** | **1.50±0.07^B^** | **1.28±0.05^A^** |  |  |  |  |
| **Toll-like receptor 4 (TLR4)** | | | | | | | | |
| **-30** | 1.00±0.00^a^ | 1.00±0.00^a^ | 1.00±0.00^a^ | 1.00±0.00^a^ | 0.155 | ≤0.001 | ≤0.001 | ns |
| **-15** | 1.49±0.13^b^ | 1.18±0.09^a^ | 1.31±0.07^bc^ | 1.12±0.15^a^ |  |  |  |  |
| **-7** | 1.68±0.10^abcB^ | 1.30±0.07^abA^ | 1.42±0.08^bcAB^ | 1.21±0.07^aA^ |  |  |  |  |
| **0** | 2.05±0.05^dC^ | 1.72±0.08^cAB^ | 1.89±0.04^dBC^ | 1.51±0.06^aA^ |  |  |  |  |
| **7** | 1.98±0.02^cdB^ | 1.62±0.09^bcAB^ | 1.87±0.05^dB^ | 1.32±0.22^aA^ |  |  |  |  |
| **15** | 1.70±0.07^bcB^ | 1.34±0.05^abA^ | 1.52±0.05^cAB^ | 1.24±0.08^aA^ |  |  |  |  |
| **30** | 1.36±0.05^b^ | 1.10±0.08^a^ | 1.20±0.11^ab^ | 1.06±0.12^a^ |  |  |  |  |
| **Overall Mean** | **1.61±0.07^D^** | **1.32±0.06^B^** | **1.46±0.07^C^** | **1.21±0.05^A^** |  |  |  |  |
| **Chemokine receptor 1 (CXCR1)** | | | | | | | | |
| **-30** | 1.00±0.00^a^ | 1.00±0.00^a^ | 1.00±0.00^a^ | 1.00±0.00^a^ | 0.113 | ≤0.001 | ≤0.001 | 0.018 |
| **-15** | 1.48±0.07^b^ | 1.36±0.07^bc^ | 1.24±0.07^ab^ | 1.21±0.07^ab^ |  |  |  |  |
| **-7** | 1.71±0.08^bcC^ | 1.59±0.04^cdeBC^ | 1.45±0.03^bcAB^ | 1.32±0.04^bcA^ |  |  |  |  |
| **0** | 2.25±0.08^dC^ | 1.93±0.08^eB^ | 1.85±0.06^dAB^ | 1.61±0.03^dA^ |  |  |  |  |
| **7** | 1.85±0.07^cB^ | 1.73±0.11^bdeAB^ | 1.69±0.05^cdAB^ | 1.44±0.05^cdA^ |  |  |  |  |
| **15** | 1.69±0.07^bcB^ | 1.51±0.10^bcdAB^ | 1.54±0.07^cAB^ | 1.29±0.03^bcA^ |  |  |  |  |
| **30** | 1.19±0.05^a^ | 1.21±0.06^ab^ | 1.17±0.08^a^ | 1.1±0.07^ab^ |  |  |  |  |
| **Overall Mean** | **1.59±0.08^C^** | **1.47±0.06^B^** | **1.42±0.06^B^** | **1.28±0.04^A^** |  |  |  |  |
| **Chemokine receptor 2 (CXCR2)** | | | | | | | | |
| **-30** | 1.00±0.00^a^ | 1.00±0.00^ab^ | 1.00±0.00^ab^ | 1.00±0.00^ab^ | 0.161 | ≤0.001 | ≤0.001 | ≤0.001 |
| **-15** | 1.12±0.06^ab^ | 0.96±0.13^a^ | 0.95±0.10^ab^ | 0.82±0.04^a^ |  |  |  |  |
| **-7** | 1.96±0.07^cC^ | 1.63±0.04^cdB^ | 1.29±0.03^bcA^ | 1.08±0.10^abA^ |  |  |  |  |
| **0** | 2.5±0.09^dC^ | 1.93±0.10^dB^ | 1.52±0.10^cAB^ | 1.42±0.11^bA^ |  |  |  |  |
| **7** | 1.46±0.11^bB^ | 1.40±0.06^bcAB^ | 1.29±0.07^bcAB^ | 1.00±0.13^A^ |  |  |  |  |
| **15** | 1.20±0.10^abB^ | 1.14±0.05^abB^ | 0.84±0.08^aAB^ | 0.73±0.07^aA^ |  |  |  |  |
| **30** | 1.15±0.05^ab^ | 1.02±0.14^ab^ | 0.88±0.14^ab^ | 0.93±0.15^a^ |  |  |  |  |
| **Overall Mean** | **1.48±0.11^C^** | **1.30±0.04^B^** | **1.11±0.06^A^** | **1.00±0.05^A^** |  |  |  |  |
| **Glucocorticoid receptors -α (GR-α)** | | | | | | | | |
| **-30** | 1.00±0.00^cd^ | 1.00±0.00^bc^ | 1.00±0.00^bcd^ | 1.00±0.00^ab^ | 0.135 | ≤0.001 | ≤0.001 | ns |
| **-15** | 0.73±0.05^bcA^ | 0.93±0.08^bAB^ | 0.82±0.04^bcA^ | 1.12±0.07^bcB^ |  |  |  |  |
| **-7** | 0.66±0.06^b^ | 0.73±0.03^ab^ | 0.69±0.07^ab^ | 0.89±0.14^ab^ |  |  |  |  |
| **0** | 0.35±0.10^aA^ | 0.49±0.05^aAB^ | 0.42±0.06^aAB^ | 0.65±0.05^aB^ |  |  |  |  |
| **7** | 0.72±0.03^bcA^ | 0.93±0.10^bAB^ | 0.88±0.04^bcA^ | 1.16±0.08^bcB^ |  |  |  |  |
| **15** | 0.93±0.06^bcd^ | 1.24±0.05^cd^ | 1.11±0.15^cd^ | 1.16±0.11^bc^ |  |  |  |  |
| **30** | 1.15±0.07^dA^ | 1.36±0.05^dAB^ | 1.26±0.09^dAB^ | 1.50±0.11^cB^ |  |  |  |  |
| **Overall Mean** | **0.79±0.05^A^** | **0.95±0.06^B^** | **0.88±0.06^AB^** | **1.06±0.06^C^** |  |  |  |  |

Means with different small superscripts (a, b, c and d) and capital superscripts (A, B and C) differ significantly (P<0.05) in a column and in a row, respectively. **RMSE:** Root Mean Square Error, **D**: Days of sampling, **Gr**: Treatment groups.

**Supplementary Table 8.** Relative mRNA expression of toll-like, chemokine and glucocorticoid receptors in blood neutrophils of calves born to different groups of periparturient Karan Fries cows, i.e. Control (Basal Diet, BD), MM (BD + Multi-mineral), MV (BD + Multi-vitamin), and MMMV (BD + Multimineral and Multivitamins).

| **Items/ Days** | **Groups** | | | | **RMSE** | **P-value** | | |
| --- | --- | --- | --- | --- | --- | --- | --- | --- |
|  | **Control** | **MM** | **MV** | **MMMV** |  | **D** | **Gr** | **D×Gr** |
| **Toll-like receptor 2 (TLR2)** | | | | | | | | |
| **0** | 1.00±0.00^c^ | 1.00±0.00^b^ | 1.00±0.00^d^ | 1.00±0.00^d^ | 0.099 | ≤0.001 | ≤0.001 | ns |
| **3** | 1.02±0.06^cB^ | 0.79±0.06^cdAB^ | 0.87±0.05^cdAB^ | 0.70±0.06^cA^ |  |  |  |  |
| **7** | 0.95±0.06^bcB^ | 0.66±0.05^bcA^ | 0.75±0.04^bcAB^ | 0.61±0.05^cA^ |  |  |  |  |
| **15** | 0.89±0.03^abcB^ | 0.55±0.07^abcA^ | 0.61±0.07^abA^ | 0.48±0.03^bcA^ |  |  |  |  |
| **30** | 0.72±0.09^abB^ | 0.47±0.05^abAB^ | 0.55±0.07^abAB^ | 0.31±0.06^abA^ |  |  |  |  |
| **45** | 0.64±0.05^aB^ | 0.36±0.07^aA^ | 0.43±0.02^aAB^ | 0.23±0.07^aA^ |  |  |  |  |
| **Overall Mean** | **0.87±0.04^C^** | **0.64±0.05^B^** | **0.70±0.05^B^** | **0.55±0.06^A^** |  |  |  |  |
| **Toll-like receptor 4 (TLR4)** | | | | | | | | |
| **0** | 1.00±0.00^b^ | 1.00±0.00^c^ | 1.00±0.00^c^ | 1.00±0.00^c^ | 0.1105 | ≤0.001 | ≤0.001 | ns |
| **3** | 0.98±0.04^b^ | 0.84±0.07^bc^ | 0.82±0.07^bc^ | 0.65±0.09^b^ |  |  |  |  |
| **7** | 0.90±0.05^b^ | 0.74±0.09^abc^ | 0.77±0.06^abc^ | 0.58±0.07^ab^ |  |  |  |  |
| **15** | 0.73±0.05^aB^ | 0.56±0.07^abAB^ | 0.62±0.09^abAB^ | 0.38±0.05^abA^ |  |  |  |  |
| **30** | 0.64±0.05^aB^ | 0.48±0.06^aAB^ | 0.52±0.08^aAB^ | 0.31±0.06^aA^ |  |  |  |  |
| **45** | 0.62±0.02^aB^ | 0.51±0.06^aAB^ | 0.51±0.02^aAB^ | 0.31±0.08^aA^ |  |  |  |  |
| **Overall Mean** | **0.81±0.04^C^** | **0.69±0.05^B^** | **0.70±0.04^B^** | **0.54±0.06^A^** |  |  |  |  |
| **Chemokine receptor 1 (CXCR1)** | | | | | | | | |
| **0** | 1.00±0.00^c^ | 1.00±0.00^d^ | 1.00±0.00^b^ | 1.00±0.00^d^ | 0.089 | ≤0.001 | ≤0.001 | ns |
| **3** | 0.9±0.05^bc^ | 0.85±0.06^cd^ | 0.86±0.05^cd^ | 0.68±0.06^c^ |  |  |  |  |
| **7** | 0.81±0.07^bc^ | 0.73±0.07^bc^ | 0.70±0.05^bc^ | 0.6±0.05^bc^ |  |  |  |  |
| **15** | 0.70±0.02^abB^ | 0.59±0.05^abAB^ | 0.56±0.06^abAB^ | 0.43±0.05^abA^ |  |  |  |  |
| **30** | 0.56±0.05^a^ | 0.51±0.06^ab^ | 0.48±0.05^a^ | 0.32±0.04^a^ |  |  |  |  |
| **45** | 0.51±0.04^aB^ | 0.44±0.05^aAB^ | 0.46±0.04^aAB^ | 0.26±0.05^aA^ |  |  |  |  |
| **Overall Mean** | **0.74±0.04^B^** | **0.68±0.05^B^** | **0.67±0.05^B^** | **0.55±0.06^A^** |  |  |  |  |
| **Chemokine receptor 2 (CXCR2)** | | | | | | | | |
| **0** | 1.00±0.00^c^ | 1.00±0.00^c^ | 1.00±0.00^d^ | 1.00±0.00^d^ | 0.114 | ≤0.001 | 0.0006 | ns |
| **3** | 0.92±0.03^c^ | 0.84±0.14^c^ | 0.80±0.04^cd^ | 0.70±0.07^c^ |  |  |  |  |
| **7** | 0.77±0.07^bc^ | 0.69±0.07^bc^ | 0.67±0.08^bc^ | 0.62±0.05^bc^ |  |  |  |  |
| **15** | 0.60±0.06^ab^ | 0.49±0.05^ab^ | 0.47±0.05^ab^ | 0.38±0.05^ab^ |  |  |  |  |
| **30** | 0.48±0.07^a^ | 0.40±0.06^ab^ | 0.37±0.07^a^ | 0.28±0.11^a^ |  |  |  |  |
| **45** | 0.44±0.04^aB^ | 0.34±0.04^aAB^ | 0.33±0.03^aAB^ | 0.19±0.07^aA^ |  |  |  |  |
| **Overall Mean** | **0.70±0.05^B^** | **0.63±0.06^B^** | **0.61±0.06^AB^** | **0.53±0.07^A^** |  |  |  |  |
| **Glucocorticoid receptors** -**α (GR-α)** | | | | | | | | |
| **0** | 1.00±0.00^a^ | 1.00±0.00^a^ | 1.00±0.00^a^ | 1.00±0.00^a^ | 0.136 | ≤0.001 | ≤0.001 | ns |
| **3** | 1.19±0.05^abA^ | 1.45±0.04^bAB^ | 1.36±0.06^bAB^ | 1.52±0.10^bB^ |  |  |  |  |
| **7** | 1.43±0.04^bcA^ | 1.66±0.03^bAB^ | 1.56±0.05^bcAB^ | 1.79±0.09^bB^ |  |  |  |  |
| **15** | 1.69±0.07^cA^ | 1.99±0.10^cA^ | 1.73±0.06^cA^ | 2.35±0.06^cB^ |  |  |  |  |
| **30** | 2.16±0.19^d^ | 2.43±0.10^d^ | 2.28±0.08^d^ | 2.55±0.07^c^ |  |  |  |  |
| **45** | 2.20±0.07^d^ | 2.50±0.09^d^ | 2.34±0.08^d^ | 2.49±0.07^c^ |  |  |  |  |
| **Overall Mean** | **1.61±0.11^A^** | **1.84±0.13^B^** | **1.71±0.11^A^** | **1.95±0.14^C^** |  |  |  |  |

Means with different small superscripts (a, b, c and d) and capital superscripts (A, B and C) differ significantly (P<0.05) in a column and in a row, respectively. **RMSE:** Root Mean Square Error, **D**: Days of sampling, **Gr**: Treatment groups.

**Supplementary Table 9.** Relative mRNA expression cluster of designation molecules in blood neutrophils of different groups of periparturient Karan Fries cows, i.e. Control (Basal Diet, BD), MM (BD + Multi-mineral), MV (BD + Multi-vitamin), and MMMV (BD + Multimineral and Multivitamins).

| **Items/ Days** | **Groups** | | | | **RMSE** | **P-value** | | |
| --- | --- | --- | --- | --- | --- | --- | --- | --- |
|  | **Control** | **MM** | **MV** | **MMMV** |  | **D** | **Gr** | **D×Gr** |
| **CD62L** | | | | | | | | |
| **-30** | 1.00±0.00^cd^ | 1.00±0.00^b^ | 1.00±0.00^abc^ | 1.00±0.00^ab^ | 0.169 | ≤0.001 | ≤0.001 | ns |
| **-15** | 0.67±0.14^abcA^ | 1.02±0.19^bAB^ | 0.97±0.15^abcAB^ | 1.25±0.10^bcAB^ |  |  |  |  |
| **-7** | 0.25±0.08^aA^ | 0.49±0.05^aB^ | 0.65±0.05^bBC^ | 0.72±0.05^aB^ |  |  |  |  |
| **0** | 0.53±0.07^abA^ | 1.13±0.06^bBC^ | 0.77±0.07^abAB^ | 1.24±0.16^bcC^ |  |  |  |  |
| **7** | 0.88±0.10^bcdA^ | 1.40±0.11^bBC^ | 1.12±0.07^bcAB^ | 1.51±0.05^cC^ |  |  |  |  |
| **15** | 1.14±0.12^d^ | 1.28±0.07^b^ | 1.04±0.13^abc^ | 1.45±0.11^c^ |  |  |  |  |
| **30** | 1.15±0.10^dA^ | 1.43±0.07^bAB^ | 1.36±0.05^cAB^ | 1.62±0.11^cB^ |  |  |  |  |
| **Overall Mean** | **0.80±0.07^A^** | **1.10±0.07^B^** | **0.99±0.05^B^** | **1.25±0.07^C^** |  |  |  |  |
| **CD11b** | | | | | | | | |
| **-30** | 1.00±0.00^a^ | 1.00±0.00^a^ | 1.00±0.00^a^ | 1.00±0.00^a^ | 0.128 | ≤0.001 | ≤0.001 | 0.0030 |
| **-15** | 1.78±0.11^bA^ | 2.23±0.09^dBC^ | 2.15±0.10^eAB^ | 2.59±0.08^dC^ |  |  |  |  |
| **-7** | 2.45±0.09^cA^ | 2.95±0.06^eB^ | 2.82±0.06^fB^ | 3.15±0.11^eB^ |  |  |  |  |
| **0** | 1.69±0.07^b^ | 1.82±0.04^c^ | 1.75±0.09^d^ | 2.00±0.05^c^ |  |  |  |  |
| **7** | 1.34±0.03^aA^ | 1.49±0.06^bA^ | 1.52±0.04^cdA^ | 1.79±0.06^bcB^ |  |  |  |  |
| **15** | 1.20±0.10^aA^ | 1.27±0.06^abA^ | 1.33±0.08^bcAB^ | 1.63±0.03^bB^ |  |  |  |  |
| **30** | 1.02±0.05^aA^ | 1.25±0.06^abA^ | 1.18±0.08^abA^ | 1.61±0.10^bB^ |  |  |  |  |
| **Overall Mean** | **1.50±0.11^A^** | **1.71±0.14^B^** | **1.68±0.13^B^** | **1.96±0.14^C^** |  |  |  |  |
| **CD25** | | | | | | | | |
| **-30** | 1.00±0.00^b^ | 1.00±0.00^bc^ | 1.00±0.00^ab^ | 1.00±0.00 | 0.988 | ≤0.001 | ≤0.001 | ns |
| **-15** | 0.85±0.03^ab^ | 0.75±0.05^ab^ | 0.82±0.04^a^ | 0.69±0.05^a^ |  |  |  |  |
| **-7** | 1.37±0.07^c^ | 1.07±0.04^c^ | 1.21±0.07^bc^ | 1.12±0.07^b^ |  |  |  |  |
| **0** | 1.56±0.06^cB^ | 1.20±0.09^cAB^ | 1.35±0.11^cAB^ | 1.09±0.08^bA^ |  |  |  |  |
| **7** | 1.00±0.07^b^ | 0.77±0.03^ab^ | 0.84±0.14^a^ | 0.66±0.02^a^ |  |  |  |  |
| **15** | 0.66±0.05^a^ | 0.69±0.07^a^ | 0.62±0.04^a^ | 0.51±0.06^a^ |  |  |  |  |
| **30** | 0.77±0.05^abB^ | 0.56±0.06^aAB^ | 0.65±0.05^aAB^ | 0.47±0.04^aA^ |  |  |  |  |
| **Overall Mean** | **1.03±0.06^C^** | **0.86±0.05^AB^** | **0.92±0.06^B^** | **0.79±0.05^A^** |  |  |  |  |
| **CD44** | | | | | | | | |
| **-30** | 1.00±0.00^a^ | 1.00±0.00^b^ | 1.00±0.00^b^ | 1.00±0.00^b^ | 0.092 | ≤0.001 | ≤0.001 | 0.005 |
| **-15** | 1.61±0.06^bB^ | 1.36±0.03^cA^ | 1.32±0.05^cA^ | 1.21±0.07^bcA^ |  |  |  |  |
| **-7** | 1.79±0.03^bB^ | 1.69±0.05^dAB^ | 1.45±0.06^cA^ | 1.43±0.08^cdA^ |  |  |  |  |
| **0** | 2.07±0.05^cB^ | 1.85±0.07^dAB^ | 1.83±0.03^dAB^ | 1.67±0.05^dA^ |  |  |  |  |
| **7** | 1.56±0.04^bB^ | 1.37±0.05^cB^ | 1.34±0.06^cB^ | 1.11±0.04^bA^ |  |  |  |  |
| **15** | 1.08±0.10^aB^ | 0.91±0.04^bB^ | 0.89±0.02^bB^ | 0.59±0.09^aA^ |  |  |  |  |
| **30** | 0.88±0.02^aC^ | 0.71±0.02^aB^ | 0.49±0.02^aA^ | 0.43±0.03^aA^ |  |  |  |  |
| **Overall Mean** | **1.42±0.09^D^** | **1.27±0.08^C^** | **1.19±0.09^B^** | **1.06±0.09^A^** |  |  |  |  |

Means with different small superscripts (a, b, c and d) and capital superscripts (A, B and C) differ significantly (P<0.05) in a column and in a row, respectively. **RMSE:** Root Mean Square Error, **D**: Days of sampling, **Gr**: Treatment groups.

**Supplementary Table 10.** Relative mRNA expression of cluster of designation molecules in blood neutrophils of calves born to different groups of periparturient Karan Fries cows, i.e. Control (Basal Diet, BD), MM (BD + Multi-mineral), MV (BD + Multi-vitamin), and MMMV (BD + Multimineral and Multivitamins).

| **Items/ Days** | **Groups** | | | | **RMSE** | **P-value** | | |
| --- | --- | --- | --- | --- | --- | --- | --- | --- |
|  | **Control** | **MM** | **MV** | **MMMV** |  | **D** | **Gr** | **D×Gr** |
| **CD62L** | | | | | | | | |
| **0** | 1.00±0.00^a^ | 1.00±0.00^a^ | 1.00±0.00^a^ | 1.00±0.00^a^ | 0.156 | ≤0.001 | ≤0.001 | ns |
| **3** | 1.07±0.04^abA^ | 1.44±0.12^bAB^ | 1.41±0.11^bAB^ | 1.72±0.11^bB^ |  |  |  |  |
| **7** | 1.38±0.04^bcA^ | 1.76±0.11^bcB^ | 1.72±0.10^bcB^ | 1.99±0.05^bB^ |  |  |  |  |
| **15** | 1.39±0.05^bcA^ | 1.82±0.09^bcB^ | 1.76±0.12^bcB^ | 2.07±0.07^bcB^ |  |  |  |  |
| **30** | 1.62±0.16^cA^ | 2.05±0.08^cAB^ | 1.98±0.08^cAB^ | 2.36±0.06^cB^ |  |  |  |  |
| **45** | 2.27±0.06^dA^ | 2.69±0.09^dAB^ | 2.64±0.11^dAB^ | 3.03±0.13^dB^ |  |  |  |  |
| **Overall Mean** | **1.45±0.10^A^** | **1.79±0.13^B^** | **1.75±0.12^B^** | **2.03±0.15^C^** |  |  |  |  |
| **CD11b** | | | | | | | | |
| **0** | 1.00±0.00^c^ | 1.00±0.00^d^ | 1.00±0.00^e^ | 1.00±0.00^d^ | 0.081 | ≤0.001 | ≤0.001 | ns |
| **3** | 0.47±0.07^bA^ | 0.58±0.05^cA^ | 0.65±0.04^dAB^ | 0.86±0.06^cdB^ |  |  |  |  |
| **7** | 0.35±0.05^abA^ | 0.41±0.07^bcA^ | 0.46±0.03^cAB^ | 0.67±0.06^bcB^ |  |  |  |  |
| **15** | 0.23±0.05^aA^ | 0.31±0.04^abAB^ | 0.39±0.04^bcAB^ | 0.48±0.06^abB^ |  |  |  |  |
| **30** | 0.15±0.02^aA^ | 0.18±0.04^aA^ | 0.24±0.03^abA^ | 0.42±0.04^aB^ |  |  |  |  |
| **45** | 0.13±0.04^aA^ | 0.15±0.02^aA^ | 0.19±0.05^aAB^ | 0.35±0.04^aB^ |  |  |  |  |
| **Overall Mean** | **0.38±0.07^A^** | **0.43±0.07^AB^** | **0.48±0.06^B^** | **0.63±0.05^C^** |  |  |  |  |
| **CD25** | | | | | | | | |
| **0** | 1.00±0.00^c^ | 1.00±0.00^d^ | 1.00±0.00^c^ | 1.00±0.00^d^ | 0.106 | ≤0.001 | ≤0.001 | ns |
| **3** | 0.94±0.15^c^ | 0.85±0.07^cd^ | 0.83±0.05^bc^ | 0.67±0.07^c^ |  |  |  |  |
| **7** | 0.84±0.10^c^ | 0.75±0.07^bc^ | 0.73±0.03^b^ | 0.58±0.05^bc^ |  |  |  |  |
| **15** | 0.75±0.04^bcC^ | 0.71±0.04^bcBC^ | 0.49±0.08^aAB^ | 0.43±0.03^bA^ |  |  |  |  |
| **30** | 0.45±0.08^abAB^ | 0.56±0.05^bB^ | 0.34±0.05^aAB^ | 0.21±0.03^aA^ |  |  |  |  |
| **45** | 0.35±0.03^aB^ | 0.31±0.02^aB^ | 0.29±0.04^aB^ | 0.06±0.03^aA^ |  |  |  |  |
| **Overall Mean** | **0.72±0.06^C^** | **0.69±0.05^BC^** | **0.61±0.06^B^** | **0.49±0.07^A^** |  |  |  |  |
| **CD44** | | | | | | | | |
| **0** | 1.00±0.00^c^ | 1.00±0.00^c^ | 1.00±0.00^c^ | 1.00±0.00^d^ | 1.258 | ≤0.001 | ≤0.001 | ≤0.001 |
| **3** | 0.79±0.07^bc^ | 0.77±0.03^bc^ | 0.84±0.03^bc^ | 0.66±0.03^c^ |  |  |  |  |
| **7** | 0.81±0.07^bc^ | 0.69±0.07^b^ | 0.62±0.07^ab^ | 0.51±0.06^bc^ |  |  |  |  |
| **15** | 0.87±0.09^bcB^ | 0.55±0.05^abA^ | 0.65±0.04^abA^ | 0.47±0.04^bcA^ |  |  |  |  |
| **30** | 0.64±0.05^abB^ | 0.41±0.05^aAB^ | 0.46±0.07^aAB^ | 0.35±0.08^abA^ |  |  |  |  |
| **45** | 0.47±0.04^a^ | 0.31±0.08^a^ | 0.39±0.07^a^ | 0.23±0.05^a^ |  |  |  |  |
| **Overall Mean** | **0.76±0.04^C^** | **0.62±0.05^B^** | **0.66±0.05^B^** | **0.53±0.06^A^** |  |  |  |  |

Means with different small superscripts (a, b, c and d) and capital superscripts (A, B and C) differ significantly (P<0.05) in a column and in a row, respectively. **RMSE:** Root Mean Square Error, **D**: Days of sampling, **Gr**: Treatment groups.

**Supplementary Table 11.** Oxidative stress biomarkers in blood plasma of different groups of periparturient Karan Fries cows, i.e. Control (Basal Diet, BD), MM (BD + Multi-mineral), MV (BD + Multi-vitamin), and MMMV (BD + Multimineral and Multivitamins).

| **Items/ Days** | **Groups** | | | | **RMSE** | **P-value** | | |
| --- | --- | --- | --- | --- | --- | --- | --- | --- |
|  | **Control** | **MM** | **MV** | **MMMV** |  | **D** | **Gr** | **D×Gr** |
| **TBARS (nmol/ml)** | | | | | | | | |
| **-30** | 7.11±0.98^a^ | 6.54±1.18^a^ | 7.081±1.13^a^ | 7.21±0.35^a^ | 1.857 | ≤0.001 | ≤0.001 | ns |
| **-15** | 8.59±0.64^a^ | 8.13±0.72^ab^ | 8.46±0.50^ab^ | 7.88±0.87^ab^ |  |  |  |  |
| **-7** | 11.77±1.21^abB^ | 8.83±0.67^abcAB^ | 9.57±0.79^abAB^ | 8.12±1.15^abA^ |  |  |  |  |
| **0** | 16.58±1.30^bcB^ | 11.88±1.30^bcdA^ | 12.52±1.33^abcA^ | 10.60±0.59^abA^ |  |  |  |  |
| **7** | 18.67±1.07^cB^ | 14.13±1.05^dAB^ | 15.60±1.69^cAB^ | 12.49±1.44^bA^ |  |  |  |  |
| **15** | 17.72±1.22^cB^ | 13.48±1.26^cdA^ | 13.89±1.01^bcA^ | 12.25±0.70^bA^ |  |  |  |  |
| **30** | 16.25±1.19^bcB^ | 12.54±0.76^bcdAB^ | 13.45±1.26^bcAB^ | 11.83±1.23^abA^ |  |  |  |  |
| **Overall Mean** | **13.81±1.02^C^** | **10.79±0.68^AB^** | **11.51±0.75^B^** | **10.05±0.56^A^** |  |  |  |  |
| **TAC (mmol/l)** | | | | | | | | |
| **-30** | 0.79±0.06^c^ | 0.79±0.06^ab^ | 0.89±0.03^c^ | 0.82±0.04^a^ | 0.114 | ≤0.001 | ≤0.001 | ns |
| **-15** | 0.66±0.05^bc^ | 0.69±0.06^ab^ | 0.64±0.04^abc^ | 0.74±0.09^a^ |  |  |  |  |
| **-7** | 0.42±0.04^abA^ | 0.62±0.04^aB^ | 0.54±0.05^abAB^ | 0.70±0.07^aB^ |  |  |  |  |
| **0** | 0.31±0.06^aA^ | 0.58±0.03^aB^ | 0.43±0.02^aAB^ | 0.65±0.11^aB^ |  |  |  |  |
| **7** | 0.42±0.03^abA^ | 0.61±0.05^aAB^ | 0.52±0.05^abAB^ | 0.75±0.08^aB^ |  |  |  |  |
| **15** | 0.57±0.09^abc^ | 0.79±0.06^ab^ | 0.71±0.06^bc^ | 0.87±0.07^aB^ |  |  |  |  |
| **30** | 0.70±0.09^bc^ | 0.89±0.07^b^ | 0.84±0.08^c^ | 0.97±0.06^a^ |  |  |  |  |
| **Overall Mean** | **0.55±0.04^A^** | **0.71±0.03^BC^** | **0.65±0.03^B^** | **0.78±0.03^C^** |  |  |  |  |
| **SOD (ng/ml)** | | | | | | | | |
| **-30** | 20.45±1.93^a^ | 21.66±2.18^a^ | 20.33±1.45^a^ | 19.61±1.78^ab^ | 3.13 | ≤0.001 | ≤0.001 | ns |
| **-15** | 24.77±2.36^ab^ | 22.00±1.52^a^ | 21.19±1.90^ab^ | 20.00±2.08^abc^ |  |  |  |  |
| **-7** | 31.29±1.61^bcB^ | 25.52±1.80^abA^ | 25.33±2.33^abcA^ | 22.63±0.79^abcA^ |  |  |  |  |
| **0** | 36.66±2.18^cB^ | 29.47±1.63^bA^ | 31.33±1.76^cAB^ | 26.52±0.51^cA^ |  |  |  |  |
| **7** | 32.66±1.87^bcB^ | 26.48±1.58^abA^ | 29.76±2.15^bcAB^ | 24.22±1.18^abcA^ |  |  |  |  |
| **15** | 31.16±2.02^bc^ | 26.03±2.33^ab^ | 27.48±1.25^abc^ | 25.52±1.69^bc^ |  |  |  |  |
| **30** | 29.16±2.60^abcC^ | 22.43±1.54^aAB^ | 27.41±1.52^abcBC^ | 18.28±1.25^aA^ |  |  |  |  |
| **Overall Mean** | **29.45±1.29^C^** | **24.80±0.83^AB^** | **26.12±1.02^B^** | **22.40±0.79^A^** |  |  |  |  |
| **CAT (ng/ml)** | | | | | | | | |
| **-30** | 15.27±1.48^ab^ | 14.32±1.27^ab^ | 12.97±0.87^a^ | 14.56±0.73^ab^ | 2.22 | ≤0.001 | ≤0.001 | ns |
| **-15** | 14.77±1.78^a^ | 14.98±1.48^ab^ | 14.22±1.13^a^ | 13.39±1.02^ab^ |  |  |  |  |
| **-7** | 20.15±1.31^abB^ | 15.85±1.24^abA^ | 17.21±1.39^abAB^ | 15.33±0.88^abA^ |  |  |  |  |
| **0** | 24.73±1.38^cB^ | 19.83±1.51^bAB^ | 22.11±1.46^bAB^ | 18.39±1.44^bA^ |  |  |  |  |
| **7** | 21.67±0.85^bcC^ | 14.75±0.70^abA^ | 17.95±1.40^abB^ | 13.96±0.70^abA^ |  |  |  |  |
| **15** | 18.32±1.41^abcB^ | 13.29±1.28^aA^ | 15.21±1.27^aAB^ | 12.23±1.36^aA^ |  |  |  |  |
| **30** | 17.84±0.76^ab^ | 16.00±1.52^ab^ | 15.4±1.70^a^ | 14.40±1.44^ab^ |  |  |  |  |
| **Overall Mean** | **18.96±0.84^C^** | **15.57±0.60^AB^** | **16.44±0.75^B^** | **14.61±0.53^A^** |  |  |  |  |

Means with different small superscripts (a, b, c and d) and capital superscripts (A, B and C) differ significantly (P<0.05) in a column and in a row, respectively. **RMSE:** Root Mean Square Error, **D**: Days of sampling, **Gr**: Treatment groups.

**Supplementary Table 12.** Oxidative stress biomarkers in blood plasma of calves born to different groups of periparturient Karan Fries cows, i.e. Control (Basal Diet, BD), MM (BD + Multi-mineral), MV (BD + Multi-vitamin), and MMMV (BD + Multimineral and Multivitamins).

| **Items/ Days** | **Groups** | | | | **RMSE** | **P-value** | | |
| --- | --- | --- | --- | --- | --- | --- | --- | --- |
|  | **Control** | **MM** | **MV** | **MMMV** |  | **D** | **Gr** | **D×Gr** |
| **TBARS (nmol/ml)** | | | | | | | | |
| **0** | 14.09±0.92^bC^ | 9.04±0.85^aAB^ | 10.88±0.36^abB^ | 8.29±0.62^abA^ | 1.261 | ≤0.001 | ≤0.001 | 0.04 |
| **3** | 16.34±1.01^bB^ | 13.35±0.48^bA^ | 13.70±1.14^bAB^ | 12.25±0.70^cA^ |  |  |  |  |
| **7** | 15.44±0.72^bB^ | 14.24±0.30^bB^ | 13.17±1.34^bAB^ | 11.01±0.35^bcA^ |  |  |  |  |
| **15** | 9.43±0.73^a^ | 8.25±0.38^a^ | 8.96±0.37^a^ | 7.75±0.24^a^ |  |  |  |  |
| **30** | 8.22±0.76^a^ | 7.35±0.17^a^ | 8.13±0.55^a^ | 7.60±1.02^a^ |  |  |  |  |
| **Overall Mean** | **12.70±0.92^C^** | **10.44±0.77^AB^** | **10.97±0.67^B^** | **9.383±0.56^A^** |  |  |  |  |
| **TAC (mmol/l)** | | | | | | | | |
| **0** | 0.30±0.04^aA^ | 0.53±0.07^aAB^ | 0.38±0.06^aAB^ | 0.59±0.05^aB^ | 0.114 | ≤0.001 | ≤0.001 | ns |
| **3** | 0.35±0.05^a^ | 0.59±0.09^a^ | 0.43±0.07^ab^ | 0.66±0.10^a^ |  |  |  |  |
| **7** | 0.41±0.04^abA^ | 0.70±0.06^aB^ | 0.53±0.07^abcAB^ | 0.72±0.02^aB^ |  |  |  |  |
| **15** | 0.64±0.05^bc^ | 0.77±0.07^a^ | 0.72±0.05^bc^ | 0.82±0.10^a^ |  |  |  |  |
| **30** | 0.73±0.05^c^ | 0.81±0.05^a^ | 0.77±0.07^c^ | 0.86±0.06^a^ |  |  |  |  |
| **Overall Mean** | **0.48±0.04^A^** | **0.68±0.04^BC^** | **0.57±0.04^AB^** | **0.73±0.03^C^** |  |  |  |  |
| **SOD (ng/ml)** | | | | | | | | |
| **0** | 24.66±1.20^a^ | 19.85±1.78^a^ | 21.32±1.84^a^ | 18.20±0.49^b^ | 2.49 | ≤0.001 | ≤0.001 | ns |
| **3** | 23.18±1.31^a^ | 18.96±1.79^a^ | 20.86±1.57^a^ | 17.41±1.15^ab^ |  |  |  |  |
| **7** | 23.52±1.47^aB^ | 16.43±1.30^aA^ | 18.45±1.71^aAB^ | 15.33±0.88^abA^ |  |  |  |  |
| **15** | 18.97±1.53^a^ | 14.28±1.71^a^ | 16.74±1.49^a^ | 14.21±1.17^ab^ |  |  |  |  |
| **30** | 19.26±1.43^aB^ | 13.59±0.84^aAB^ | 16.48±1.50^aAB^ | 12.33±1.66^aA^ |  |  |  |  |
| **Overall Mean** | **21.92±0.81^C^** | **16.62±0.87^AB^** | **18.77±0.81^B^** | **15.50±0.71^A^** |  |  |  |  |
| **Catalase (ng/ml)** | | | | | | | | |
| **0** | 19.96±0.75^cB^ | 15.51±0.45^cA^ | 16.38±0.78^cA^ | 15.16±0.94^cA^ | 1.599 | ≤0.001 | ≤0.001 | ns |
| **3** | 19.07±0.96^bcB^ | 15.33±0.33^cA^ | 15.74±0.93^cAB^ | 13.24±0.71^cA^ |  |  |  |  |
| **7** | 15.62±0.85^bB^ | 12.44±0.96^bcAB^ | 14.88±0.94^bcAB^ | 11.38±0.91^bcA^ |  |  |  |  |
| **15** | 10.90±1.07^a^ | 9.43±0.63^ab^ | 10.70±1.24^ab^ | 8.40±0.67^ab^ |  |  |  |  |
| **30** | 9.49±0.92^a^ | 7.46±1.89^a^ | 8.93±0.61^a^ | 7.25±0.77^a^ |  |  |  |  |
| **Overall Mean** | **15.01±1.17^C^** | **12.03±0.93^AB^** | **13.32±0.86^BC^** | **11.08±0.84^A^** |  |  |  |  |

Means with different small superscripts (a, b and c) and capital superscripts (A, B and C) differ significantly (P<0.05) in a column and in a row, respectively. **RMSE:** Root Mean Square Error, **D**: Days of sampling, **Gr**: Treatment groups.

**Supplementary Table 13.** The concentration of blood plasma pro-inflammatory cytokines (IL-1α, IL-1β, IL-6 and IL-8) of different groups of periparturient Karan Fries cows, i.e. Control (Basal Diet, BD), MM (BD + Multi-mineral), MV (BD + Multi-vitamin), and MMMV (BD + Multimineral and Multivitamins).

| **Items/ Days** | **Groups** | | | | **RMSE** | **P-value** | | |
| --- | --- | --- | --- | --- | --- | --- | --- | --- |
|  | **Control** | **MM** | **MV** | **MMMV** |  | **D** | **Gr** | **D×Gr** |
| **IL-1α (pg/ml)** | | | | | | | | |
| **-30** | 32.00±2.08^b^ | 32.00±2.30^bc^ | 35.00±2.64^bc^ | 30.00±1.52^bc^ | 5.08 | ≤0.001 | ≤0.001 | ns |
| **-15** | 45.00±3.60^c^ | 40.00±2.51^cd^ | 42.00±2.64^cd^ | 38.00±2.30^cd^ |  |  |  |  |
| **-7** | 53.00±2.30^c^ | 48.00±1.73^de^ | 50.00±2.64^d^ | 46.00±3.21^d^ |  |  |  |  |
| **0** | 68.00±2.08^dB^ | 53.00±4.16^eA^ | 55.00±4.93^dA^ | 50.00±4.35^dA^ |  |  |  |  |
| **7** | 32.00±2.64^b^ | 28.00±2.64^bc^ | 29.00±4.61^abc^ | 25.00±2.51^ab^ |  |  |  |  |
| **15** | 23.00±3.21^ab^ | 20.00±3.78^ab^ | 22.00±2.64^ab^ | 18.00±2.64^ab^ |  |  |  |  |
| **30** | 18.00±2.08^a^ | 15.00±2.00^a^ | 16.00±2.08^a^ | 15.00±2.64^a^ |  |  |  |  |
| **Overall Mean** | **38.71±3.74^B^** | **33.71±3.05^AB^** | **35.57±3.15^AB^** | **31.71±2.93^A^** |  |  |  |  |
| **IL-1β (pg/ml)** | | | | | | | | |
| **-30** | 300.00±21.50^bc^ | 290.00±19.55^bc^ | 310.66±16.04^b^ | 317.00±22.50^b^ | 28.92 | ≤0.001 | ≤0.001 | ns |
| **-15** | 347.00±23.43^c^ | 330.00±20.03^c^ | 329.66±9.95^b^ | 320.33±18.40^b^ |  |  |  |  |
| **-7** | 337.33±21.60^c^ | 321.33±12.20^c^ | 319.66±16.38^b^ | 300.00±.20.66^b^ |  |  |  |  |
| **0** | 460.00±16.07^dB^ | 400.00±18.47^dA^ | 420.66±15.07^cAB^ | 380.00±18.06^bA^ |  |  |  |  |
| **7** | 277.33±16.59^abcB^ | 230.33±15.60^abAB^ | 207.00±14.74^aA^ | 200.33±14.74^aA^ |  |  |  |  |
| **15** | 250.33±9.06^abC^ | 190.00±8.08^aAB^ | 220.33±13.54^aBC^ | 180.00±13.61^aA^ |  |  |  |  |
| **30** | 200.00±17.08^a^ | 180.00±13.11^a^ | 202.66±8.76^a^ | 210.00±18.33^a^ |  |  |  |  |
| **Overall Mean** | **310.28±18.16^B^** | **277.38±17.47^A^** | **287.23±17.30^A^** | **272.52±16.67^A^** |  |  |  |  |
| **IL-6 (pg/ml)** | | | | | | | | |
| **-30** | 394.33±19.54^cA^ | 496.66±26.19^cB^ | 360.00±20.00^cA^ | 373.66±11.83^bA^ | 35.76 | ≤0.001 | ≤0.001 | ≤0.001 |
| **-15** | 741.33±26.74^dC^ | 586.66±20.27^dB^ | 489.00±14.64^dA^ | 510.33±26.90^cA^ |  |  |  |  |
| **-7** | 1120.00±35.11^fD^ | 790.00±28.43^eB^ | 920.00±17.32^fC^ | 628.33±14.81^eA^ |  |  |  |  |
| **0** | 1299.33±24.77^gC^ | 955.00±24.66^fA^ | 1204.00±29.14^gB^ | 997.66±22.55^eA^ |  |  |  |  |
| **7** | 1004.33±33.94^eC^ | 516.66±14.52^cdAB^ | 588.00±15.62^eB^ | 493.00±27.00^cA^ |  |  |  |  |
| **15** | 194.66±13.19^bB^ | 256.66±7.26^bC^ | 211.00±14.64^bB^ | 143.33±9.38^aA^ |  |  |  |  |
| **30** | 75.66±7.88^a^ | 88.00±10.06^a^ | 73.00±6.42^a^ | 72.66±7.21^a^ |  |  |  |  |
| **Overall Mean** | **689.95±99.15^C^** | **527.09±61.44^B^** | **549.28±82.49^B^** | **459.85±64.57^A^** |  |  |  |  |
| **IL-8 (pg/ml)** | | | | | | | | |
| **-30** | 155.60±10.58^aA^ | 140.00±7.63^aAB^ | 166.00±6.24^abB^ | 130.60±8.08^aA^ | 19.18 | ≤0.001 | ≤0.001 | 0.0131 |
| **-15** | 262.00±13.31^cdB^ | 202.00±9.60^bA^ | 211.30±10.39^bA^ | 190.00±10.78^bA^ |  |  |  |  |
| **-7** | 290.30±15.60^dAB^ | 295.00±7.63^dB^ | 289.30±10.17^cAB^ | 255.00±5.77^cA^ |  |  |  |  |
| **0** | 523.30±17.63^eB^ | 472.60±10.10^eA^ | 460.30±15.76^dA^ | 427.00±9.53^dA^ |  |  |  |  |
| **7** | 295.00±13.22^dC^ | 254.00±6.24^cB^ | 277.00±13.00^cBC^ | 203.00±12.50^bA^ |  |  |  |  |
| **15** | 215.00±8.66^bcB^ | 211.30±17.03^bB^ | 213.30±11.66^bB^ | 140.60±10.74^aA^ |  |  |  |  |
| **30** | 166.30±8.41^abC^ | 130.00±5.77^aAB^ | 155.30±11.40^aBC^ | 106.00±9.16^aA^ |  |  |  |  |
| **Overall Mean** | **272.52±25.96^C^** | **243.57±24.30^B^** | **253.23±21.90^B^** | **207.47±22.79^A^** |  |  |  |  |

Means with different small superscripts (a, b, c and d) and capital superscripts (A, B and C) differ significantly (P<0.05) in a column and in a row, respectively. **RMSE:** Root Mean Square Error, **D**: Days of sampling, **Gr**: Treatment groups.

**Supplementary Table 14.** The concentration of blood plasma pro-inflammatory cytokines (IL-1α, IL-1β, IL-6 and IL-8) in calves born to different groups of periparturient Karan Fries cows, i.e. Control (Basal Diet, BD), MM (BD + Multi-mineral), MV (BD + Multi-vitamin), and MMMV (BD + Multimineral and Multivitamins).

| **Items/ Days** | **Groups** | | | | **RMSE** | **P-value** | | |
| --- | --- | --- | --- | --- | --- | --- | --- | --- |
|  | **Control** | **MM** | **MV** | **MMMV** |  | **D** | **Gr** | **D×Gr** |
| **IL-1α (pg/ml)** | | | | | | | | |
| **0** | 51.00±4.16^c^ | 45.00±2.88^d^ | 48.00±4.35^d^ | 40.00±3.46^b^ | 5.17 | ≤0.001 | 0.0023 | ns |
| **3** | 35.00±3.60^b^ | 35.00±3.60^cd^ | 33.00±2.51^c^ | 32.00±3.60^b^ |  |  |  |  |
| **7** | 32.00±3.05^abB^ | 24.00±3.05^bcAB^ | 26.00±3.00^bcAB^ | 20.00±2.51^aA^ |  |  |  |  |
| **15** | 21.00±2.64^abB^ | 11.00±2.30^aA^ | 14.00±1.15^aA^ | 15.00±1.52^aAB^ |  |  |  |  |
| **30** | 18.00±3.51^a^ | 16.00±2.00^ab^ | 18.00±1.73^ab^ | 12.00±2.64^a^ |  |  |  |  |
| **Overall Mean** | **31.40±3.38^B^** | **26.20±3.48^A^** | **27.80±3.38^AB^** | **23.80±3.02^A^** |  |  |  |  |
| **IL-1β (pg/ml)** | | | | | | | | |
| **0** | 600.66±27.22^dB^ | 550.66±16.14^dAB^ | 610.00±24.19^dB^ | 500.66±18.47^dA^ | 28.83 | ≤0.001 | ≤0.001 | ns |
| **3** | 400.33±10.68^cB^ | 332.00±14.10^cA^ | 340.33±19.16^cA^ | 310.33±16.18^cA^ |  |  |  |  |
| **7** | 300.00±18.77^bB^ | 190.66±24.60^bA^ | 210.66±11.66^bA^ | 200.66±18.09^bA^ |  |  |  |  |
| **15** | 220.33±21.67^aC^ | 150.33±9.76^abAB^ | 200.66±14.81^bBC^ | 130.33±8.37^aA^ |  |  |  |  |
| **30** | 172.00±12.16^aB^ | 100.00±10.40^aA^ | 90.33±7.31^aA^ | 120.33±9.52^aA^ |  |  |  |  |
| **Overall Mean** | **338.66±41.23^B^** | **264.7±43.80^A^** | **290.44±48.00^AB^** | **252.46±38.20^A^** |  |  |  |  |
| **IL-6 (pg/ml)** | | | | | | | | |
| **0** | 177.00±9.29^cB^ | 117.00±8.50^cA^ | 111.00±9.53^dA^ | 85.00±5.29^cA^ | 8.65 | ≤0.001 | ≤0.001 | ≤0.001 |
| **3** | 87.00±4.00^bB^ | 66.00±3.51^bA^ | 64.00±2.30^cA^ | 52.66±3.71^bA^ |  |  |  |  |
| **7** | 40.66±4.70^a^ | 42.33±4.09^ab^ | 52.66±5.54^bc^ | 36.66±2.02^a^ |  |  |  |  |
| **15** | 24.00±2.08^aA^ | 30.66±2.02^aA^ | 24.33±2.60^aA^ | 44.33±5.20^aB^ |  |  |  |  |
| **30** | 32.66±3.52^a^ | 30.00±3.78^a^ | 31.00±4.93^ab^ | 28.00±2.64^a^ |  |  |  |  |
| **Overall Mean** | **71.33±15.46^B^** | **54.53±8.83^A^** | **56.60±8.48^A^** | **52.93±5.49^A^** |  |  |  |  |
| **IL-8 (pg/ml)** | | | | | | | | |
| **0** | 521.66±32.18^cB^ | 441.33±14.85^dA^ | 478.00±11.93^dAB^ | 416.00±11.26^dA^ | 21.85 | ≤0.001 | ≤0.001 | 0.0038 |
| **3** | 256.66±8.81^bB^ | 236.00±13.45^cB^ | 246.00±19.39^bB^ | 181.30±14.09^cA^ |  |  |  |  |
| **7** | 198.33±10.13^bB^ | 161.00±15.63^bAB^ | 181.00±9.60^bB^ | 127.00±7.63^bA^ |  |  |  |  |
| **15** | 118.00±10.78^aC^ | 76.00±6.65^aB^ | 42.00±5.68^aA^ | 71.66±6.35^aAB^ |  |  |  |  |
| **30** | 66.00±3.21^a^ | 61.00±5.29^a^ | 59.00±6.55^a^ | 56.00±3.21^a^ |  |  |  |  |
| **Overall Mean** | **232.10±42.90^C^** | **195.00±37.20^A^** | **201.20±42.42^B^** | **170.40±35.05^A^** |  |  |  |  |

Means with different small superscripts (a, b, c and d) and capital superscripts (A, B and C) differ significantly (P<0.05) in a column and in a row, respectively. **RMSE:** Root Mean Square Error, **D**: Days of sampling, **Gr**: Treatment groups.

**Supplementary Table 15.** The concentration of blood plasma pro-inflammatory cytokines (IL-17A, TNF-α and IFN-γ) of different groups of periparturient Karan Fries cows, i.e. Control (Basal Diet, BD), MM (BD + Multi-mineral), MV (BD + Multi-vitamin), and MMMV (BD + Multimineral and Multivitamins).

| **Items/ Days** | **Groups** | | | | **RMSE** | **P-value** | | |
| --- | --- | --- | --- | --- | --- | --- | --- | --- |
|  | **Control** | **MM** | **MV** | **MMMV** |  | **D** | **Gr** | **D×Gr** |
| **IL-17A (pg/ml)** | | | | | | | | |
| **-30** | 45.33±5.69^a^ | 43.00±4.04^ab^ | 46.33±8.76^ab^ | 44.00±4.50^ab^ | 10.37 | ≤0.001 | ≤0.001 | ns |
| **-15** | 53.00±7.44^ab^ | 46.67±5.33^ab^ | 51.34±5.85^ab^ | 49.63±6.45^ab^ |  |  |  |  |
| **-7** | 72.66±5.89^b^ | 58.00±3.60^bc^ | 66.00±6.65^bc^ | 55.00±8.50^ab^ |  |  |  |  |
| **0** | 95.66±4.80^c^ | 74.00±7.09^c^ | 81.00±5.29^c^ | 71.00±7.63^b^ |  |  |  |  |
| **7** | 51.00±5.84^ab^ | 33.30±3.06^a^ | 42.67±5.20^ab^ | 31.67±7.31^a^ |  |  |  |  |
| **15** | 41.00±4.91^a^ | 28.34±3.51^a^ | 38.34±5.68^ab^ | 26.00±7.44^a^ |  |  |  |  |
| **30** | 38.33±2.90^a^ | 26.00±4.50^a^ | 28.66±8.21^a^ | 29.00±5.56^a^ |  |  |  |  |
| **Overall Mean** | **56.71±4.59^B^** | **44.18±3.86^A^** | **50.62±4.22^AB^** | **43.75±4.00^A^** |  |  |  |  |
| **TNF-α (pg/ml)** | | | | | | | | |
| **-30** | 287.00±19.08^a^ | 255.33±18.09^a^ | 284.48±15.30^a^ | 270.33±16.04^a^ | 37.62 | ≤0.001 | ≤0.001 | ns |
| **-15** | 339.00±26.65^abA^ | 270.99±20.69^aAB^ | 290.00±19.85^aAB^ | 239.99±11.79^aB^ |  |  |  |  |
| **-7** | 839.00±36.89^dC^ | 529.00±13.20^bA^ | 628.66±26.96^cB^ | 511.33±18.76^bA^ |  |  |  |  |
| **0** | 936.67±31.79^eC^ | 809.33±27.76^dB^ | 838.00±31.00^dB^ | 677.42±25.16^cA^ |  |  |  |  |
| **7** | 697.33±18.88^cA^ | 675.66±21.07^cA^ | 630.00±24.33^cA^ | 646.33±18.55^cB^ |  |  |  |  |
| **15** | 626.00±19.85^cC^ | 522.66±13.66^bB^ | 382.33±23.80^bA^ | 477.66±17.89^bB^ |  |  |  |  |
| **30** | 400.00±15.01^bB^ | 255.36±18.46^aA^ | 209.66±14.31^aA^ | 218.11±19.75^aA^ |  |  |  |  |
| **Overall Mean** | **589.28±53.10^C^** | **474.05±46.34^B^** | **466.16±48.97^B^** | **434.45±40.36^A^** |  |  |  |  |
| **IFN-γ (pg/ml)** | | | | | | | | |
| **-30** | 40.00±5.77^a^ | 53.66±4.09^a^ | 48.54±6.62^a^ | 56.51±4.53^a^ | 13.63 | ≤0.001 | ≤0.001 | ≤0.001 |
| **-15** | 72.00±6.08^a^ | 60.00±5.29^a^ | 70.65±2.86^ab^ | 62.08±2.14 |  |  |  |  |
| **-7** | 140.00±6.92^bB^ | 120.33±5.84^cAB^ | 150.33±13.20^cB^ | 102.00±10.59^bA^ |  |  |  |  |
| **0** | 281.67±17.42^eB^ | 254.33±6.98^dAB^ | 250.63±11.90^dAB^ | 228.33±9.82^dA^ |  |  |  |  |
| **7** | 224.67±8.66^dC^ | 139.66±7.88^cA^ | 180.66±5.92^cB^ | 163.00±9.50^cAB^ |  |  |  |  |
| **15** | 184.33±9.93^cC^ | 84.33±6.17^bAB^ | 101.00±6.00^bB^ | 63.66±2.00^aA^ |  |  |  |  |
| **30** | 54.00±6.42^a^ | 36.00±4.04^a^ | 49.66±6.38^a^ | 42.66±6.69^a^ |  |  |  |  |
| **Overall Mean** | **142.38±19.35^C^** | **106.90±15.59^A^** | **121.64±15.91^B^** | **102.61±14.41^A^** |  |  |  |  |

Means with different small superscripts (a, b, c and d) and capital superscripts (A, B and C) differ significantly (P<0.05) in a column and in a row, respectively. **RMSE:** Root Mean Square Error, **D**: Days of sampling, **Gr**: Treatment groups.

**Supplementary Table 16.** The concentration of blood plasma pro-inflammatory cytokines (IL-17A, TNF-α and IFN-γ) in calves born to different groups of periparturient Karan Fries cows, i.e. Control (Basal Diet, BD), MM (BD + Multi-mineral), MV (BD + Multi-vitamin), and MMMV (BD + Multimineral and Multivitamins).

| **Items/ Days** | **Groups** | | | | **RMSE** | **P-value** | | |
| --- | --- | --- | --- | --- | --- | --- | --- | --- |
|  | **Control** | **MM** | **MV** | **MMMV** |  | **D** | **Gr** | **D×Gr** |
| **IL-17A (pg/ml)** | | | | | | | | |
| **0** | 19.00±2.88^b^ | 14.66±2.60^b^ | 15.33±2.02^c^ | 14.66±1.33^c^ | 2.37 | ≤0.001 | ns | ns |
| **3** | 9.66±2.18^a^ | 8.33±1.33^a^ | 9.66±1.20^b^ | 6.66±0.88^b^ |  |  |  |  |
| **7** | 6.66±1.20^a^ | 5.66±1.20^a^ | 6.00±1.15^ab^ | 5.00±0.57^a^ |  |  |  |  |
| **15** | 4.00±0.57^a^ | 4.66±0.66^a^ | 4.66±0.66^ab^ | 4.00±0.57^ab^ |  |  |  |  |
| **30** | 3.33±0.88^a^ | 2.66±0.66^a^ | 3.00±0.57^a^ | 1.66±0.33^a^ |  |  |  |  |
| **Overall Mean** | **8.53±1.66** | **7.20±1.24** | **7.73±1.26** | **6.4±1.22** |  |  |  |  |
| **TNF-α (pg/ml)** | | | | | | | | |
| **0** | 254.88±16.28^dB^ | 179.16±12.23^cA^ | 234.66±13.92^cBC^ | 190.33±14.62^cAB^ | 17.07 | ≤0.001 | ≤0.001 | 0.022 |
| **3** | 146.00±9.71^c^ | 126.33±15.62^b^ | 134.79±6.24^b^ | 112.00±10.44^b^ |  |  |  |  |
| **7** | 88.00±6.35^bB^ | 68.33±4.48^aAB^ | 63.72±14.25^aAB^ | 43.30±5.29^aA^ |  |  |  |  |
| **15** | 89.47±12.14^bB^ | 50.00±3.51^aA^ | 66.29±7.31^aAB^ | 54.00±4.58^aA^ |  |  |  |  |
| **30** | 38.00±4.25^a^ | 39.00±6.24^a^ | 33.11±3.94^a^ | 41.00±6.02^a^ |  |  |  |  |
| **Overall Mean** | **123.27±15.22^B^** | **92.56±14.55^AB^** | **106.51±19.66^AB^** | **88.12±15.68^A^** |  |  |  |  |
| **IFN-γ (pg/ml)** | | | | | | | | |
| **0** | 54.33±5.81^c^ | 41.66±3.84^b^ | 46.33±4.37^c^ | 41.33±4.33^c^ |  |  |  |  |
| **3** | 35.00±5.19^bc^ | 34.33±5.81^b^ | 31.66±3.84^b^ | 29.00±4.58^b^ |  |  |  |  |
| **7** | 23.66±3.48^abB^ | 16.74±1.49^aAB^ | 18.00±2.30^aAB^ | 12.63±2.63^aA^ |  |  |  |  |
| **15** | 20.13±2.05^abB^ | 13.67±0.84^aAB^ | 15.20±2.11^aAB^ | 12.12±1.06^aA^ |  |  |  |  |
| **30** | 12.66±1.85^aB^ | 8.01±0.14^aA^ | 11.24±0.74^aAB^ | 10.23±0.27^aAB^ |  |  |  |  |
| **Overall Mean** | **29.16±2.16^B^** | **22.88±3.64^AB^** | **24.48±3.62^AB^** | **21.06±3.46^A^** |  |  |  |  |

Means with different small superscripts (a, b, c and d) and capital superscripts (A, B and C) differ significantly (P<0.05) in a column and in a row, respectively. **RMSE:** Root Mean Square Error, **D**: Days of sampling, **Gr**: Treatment groups.

**Supplementary Table 17.** The concentration of blood plasma anti-inflammatory cytokines (IL-4 and IL-10) of different groups of periparturient Karan Fries cows, i.e. Control (Basal Diet, BD), MM (BD + Multi-mineral), MV (BD + Multi-vitamin), and MMMV (BD + Multimineral and Multivitamins).

| **Items/ Days** | **Groups** | | | | **RMSE** | **P-value** | | |
| --- | --- | --- | --- | --- | --- | --- | --- | --- |
|  | **Control** | **MM** | **MV** | **MMMV** |  | **D** | **Gr** | **D×Gr** |
| **IL-4 (pg/ml)** | | | | | | | | |
| **-30** | 580.00±37.85^f^ | 559.33±31.83^e^ | 520.00±22.94^f^ | 568.00±22.89^e^ | 29.36 | ≤0.001 | ≤0.001 | 0.019 |
| **-15** | 328.00±19.97^eA^ | 405.00±10.44^dB^ | 328.66±35.26^eA^ | 474.00±14.00^dB^ |  |  |  |  |
| **-7** | 245.00±12.09^dA^ | 289.00±8.50^cAB^ | 275.00±11.15^dAB^ | 305.00±19.07^cB^ |  |  |  |  |
| **0** | 98.00±4.72^abA^ | 125.00±3.78^aB^ | 111.00±8.32^abAB^ | 128.00±8.50^aB^ |  |  |  |  |
| **7** | 69.00±6.80^aA^ | 90.00±6.65^aAB^ | 85.00±10.40^aAB^ | 110.00±11.78^aB^ |  |  |  |  |
| **15** | 150.00±10.11^bcA^ | 195.66±20.07^bB^ | 173.00±6.24^bcAB^ | 210.00±13.07^bB^ |  |  |  |  |
| **30** | 185.00±11.26^cdA^ | 234.00±14.36^bcB^ | 211.00±9.71^cdAB^ | 248.00±15.39^bcB^ |  |  |  |  |
| **Overall Mean** | **236.42±36.68** | **271.14±34.46** | **243.38±31.37** | **291.85±36.01** |  |  |  |  |
| **IL-10 (pg/ml)** | | | | | | | | |
| **-30** | 941.33±21.30^dA^ | 960.66±30.33^dAB^ | 960.00±20.81^dAB^ | 1040.00±32.14^cB^ | 46.95 | ≤0.001 | ≤0.001 | ≤0.001 |
| **-15** | 800.00±23.62^cA^ | 816.66±21.32^bcAB^ | 784±20.59^cA^ | 880.33±11.25^bB^ |  |  |  |  |
| **-7** | 629.33±35.87^bA^ | 728.33±20.00^bBC^ | 656.00±18.58^bAB^ | 792.00±17.78^bC^ |  |  |  |  |
| **0** | 381.66±11.31^aA^ | 520.00±16.74^aB^ | 416.33±18.41^aA^ | 568.33±27.76^aB^ |  |  |  |  |
| **7** | 359.00±22.14^aA^ | 405.33±20.00^aA^ | 368.00±20.13^aA^ | 474.00±13.05^aB^ |  |  |  |  |
| **15** | 800.00±24.21^cA^ | 931.00±31.00^cdB^ | 952.00±22.81^dBC^ | 1035.60±25.62^cC^ |  |  |  |  |
| **30** | 978.33±50.60^dA^ | 1413.33±58.11^eB^ | 964.66±23.18^dA^ | 1365.6±40.48^dB^ |  |  |  |  |
| **Overall Mean** | **698.52±52.80^A^** | **825.04±39.07^B^** | **728.71±33.52^A^** | **879.42±43.51^B^** |  |  |  |  |

Means with different small superscripts (a, b, c and d) and capital superscripts (A, B and C) differ significantly (P<0.05) in a column and in a row, respectively. **RMSE:** Root Mean Square Error, **D**: Days of sampling, **Gr**: Treatment groups.

**Supplementary Table 18.** The concentration of blood plasma anti-inflammatory cytokines (IL-4 and IL-10) in calves born to different groups of periparturient Karan Fries cows, i.e. Control (Basal Diet, BD), MM (BD + Multi-mineral), MV (BD + Multi-vitamin), and MMMV (BD + Multimineral and Multivitamins).

| **Items/ Days** | **Groups** | | | | **RMSE** | **P-value** | | |
| --- | --- | --- | --- | --- | --- | --- | --- | --- |
|  | **Control** | **MM** | **MV** | **MMMV** |  | **D** | **Gr** | **D×Gr** |
| **IL-4 (pg/ml)** | | | | | | | | |
| **0** | 29.00±3.21^aA^ | 40.00±3.21^aB^ | 35.00±3.78^aAB^ | 52.00±2.08^aC^ | 7.60 | ≤0.001 | ≤0.001 | ns |
| **3** | 44.00±7.00^aA^ | 66.00±3.78^bB^ | 58.00±4.04^bAB^ | 72.00±1.52^bB^ |  |  |  |  |
| **7** | 43.66±4.97^aA^ | 68.33±3.17^bB^ | 65.00±3.60^bB^ | 75.00±2.08^bB^ |  |  |  |  |
| **15** | 87.00±5.29^b^ | 93.00±2.30^c^ | 89.00±4.72^c^ | 97.00±4.72^c^ |  |  |  |  |
| **30** | 110.00±8.71^b^ | 113.00±4.72^d^ | 107.00±4.93^d^ | 120.00±3.78^d^ |  |  |  |  |
| **Overall Mean** | **62.73±8.49^A^** | **76.06±6.80^AB^** | **70.80±6.86^AB^** | **83.20±6.33^B^** |  |  |  |  |
| **IL-10 (pg/ml)** | | | | | | | | |
| **0** | 31.66±5.48^aA^ | 51.33±5.20^aA^ | 48.00±6.24^aB^ | 74.33±9.38^aB^ | 18.30 | ≤0.001 | ≤0.001 | ns |
| **3** | 54.33±6.98^aA^ | 75.00±8.71^aAB^ | 65.33±6.06^aAB^ | 91.66±9.13^aB^ |  |  |  |  |
| **7** | 94.33±8.66^bA^ | 123.33±11.25^bAB^ | 103.00±11.35^bA^ | 127.33±13.66^aB^ |  |  |  |  |
| **15** | 170.33±9.95^c^ | 189.33±10.39^c^ | 194.00±7.21^c^ | 185.33±16.90^b^ |  |  |  |  |
| **30** | 185.00±6.08^cA^ | 221.00±12.8^cB^ | 205.00±10.96^cAB^ | 209.33±20.49^bAB^ |  |  |  |  |
| **Overall Mean** | **107.13±10.59^A^** | **132.00±17.74^AB^** | **123.06±17.67^AB^** | **137.60±15.00^B^** |  |  |  |  |

Means with different small superscripts (a, b, c and d) and capital superscripts (A, B and C) differ significantly (P<0.05) in a column and in a row, respectively. **RMSE:** Root Mean Square Error, **D**: Days of sampling, **Gr**: Treatment groups.

**Supplementary Table 19.** Total immunoglobulins (TIG) (mg/ml) concentration in colostrum/milk whey of different groups of periparturient Karan Fries cows, i.e. Control (Basal Diet, BD), MM (BD + Multi-mineral), MV (BD + Multi-vitamin), and MMMV (BD + Multimineral and Multivitamins).

| **Items/ Days** | **Groups** | | | | **RMSE** | **P-value** | | |
| --- | --- | --- | --- | --- | --- | --- | --- | --- |
|  | **Control** | **MM** | **MV** | **MMMV** |  | **D** | **Gr** | **D×Gr** |
| **Total immunoglobulins (TIG) (mg/ml)** | | | | | | | | |
| **0** | 75.16±5.46^cA^ | 86.08±5.22^dA^ | 81.50±3.25^dA^ | 104.41±8.09^cB^ | 2.85 | ≤0.001 | ≤0.001 | 0.014 |
| **2** | 25.91±3.46^bA^ | 32.16±2.16 ^cAB^ | 28.81±1.65^cAB^ | 35.43±3.18^bB^ |  |  |  |  |
| **4** | 11.18±1.75^a^ | 14.28±1.87 ^b^ | 12.17±0.40^b^ | 15.59±2.09^a^ |  |  |  |  |
| **8** | 0.305±0.04^a^ | 0.418±0.03 ^a^ | 0.511±0.14^a^ | 0.66±0.12^a^ |  |  |  |  |
| **Overall Mean** | **28.14±4.74^A^** | **33.23±9.88^AB^** | **30.74±9.37^AB^** | **39.02±7.10^B^** |  |  |  |  |

Means with different small superscripts (a, b, c and d) and capital superscripts (A, and B) differ significantly (P<0.05) in a column and in a row, respectively. **RMSE:** Root Mean Square Error, **D**: Days of sampling, **Gr**: Treatment groups.

**Supplementary Table 20.** Total immunoglobulins (TIG) (mg/ml) concentration in blood plasma of calves born to different groups of periparturient Karan Fries cows, i.e. Control (Basal Diet, BD), MM (BD + Multi-mineral), MV (BD + Multi-vitamin), and MMMV (BD + Multimineral and Multivitamins).

| **Items/ Days** | **Groups** | | | | **RMSE** | **P-value** | | |
| --- | --- | --- | --- | --- | --- | --- | --- | --- |
|  | **Control** | **MM** | **MV** | **MMMV** |  | **D** | **Gr** | **D×Gr** |
| **Total immunoglobulins (TIG) (mg/ml)** | | | | | | | | |
| **1** | 21.70±1.83^bA^ | 29.06±1.87^bB^ | 23.12±1.51^bA^ | 34.32±1.85^bC^ | 2.24 | ≤0.001 | ≤0.001 | 0.04 |
| **2** | 19.83±1.25^bA^ | 27.5±1.37^bB^ | 20.79±1.42^bA^ | 33.16±1.26^bC^ |  |  |  |  |
| **4** | 14.91±1.02^aA^ | 17.45±0.68^aA^ | 15.56±1.21^aA^ | 22.33±1.31^aB^ |  |  |  |  |
| **8** | 11.40±0.29^aA^ | 14.12±1.14^aA^ | 12.57±0.62^aA^ | 17.15±0.76^aB^ |  |  |  |  |
| **Overall Mean** | **16.96±1.33^A^** | **22.03±2.00^BC^** | **18.01±1.36^AB^** | **26.74±2.25^C^** |  |  |  |  |

Means with different small superscripts (a, and b) and capital superscripts (A, B and C) differ significantly (P<0.05) in a column and in a row, respectively. **RMSE:** Root Mean Square Error, **D**: Days of sampling, **Gr**: Treatment groups.
